# Association Between Joint Commission International Patient‐Centered Standards and Self‐Reported Nursing Performance in Sana′a, Yemen Hospitals

**DOI:** 10.1155/jonm/8353270

**Published:** 2026-05-30

**Authors:** Kamal Ahmed Qabban, Muneer Musleh Al-Wesabi, Haitham Mohammed Jowah

**Affiliations:** ^1^ Center of Business Administration, Sana′a University, Sana′a, Yemen, su.edu.ye; ^2^ 21 September University for Medical and Applied Sciences, Sana’a, Yemen; ^3^ Department of Surgery, Faculty of Medicine and Health Sciences, Sana’a University, Sana’a, Yemen, su.edu.ye

**Keywords:** cross-sectional study, healthcare quality, hospital accreditation, JCI standards, measurement invariance, nursing leadership, nursing management, patient-centered care, self-reported nursing performance

## Abstract

**Background:**

Yemen’s prolonged conflict has severely strained the healthcare infrastructure, creating resource shortages and staffing deficits that compromise nursing practice and patient safety. The Joint Commission International (JCI) provides globally recognized patient‐centered standards for quality improvement; however, their implementation and association with self‐reported nursing performance remain underexplored in fragile, resource‐constrained settings such as Yemen.

**Methods:**

This cross‐sectional study was conducted between August and October 2024 at six hospitals in Sana′a, Yemen. A simple random sample of 526 nurses from emergency, inpatient, intensive care, and neonatal units completed a validated 66‐item questionnaire assessing six JCI domains—international patient safety goals (IPSGs), access to care and continuity (ACC), patient‐centered care (PCC), assessment of patients (AOP), care of patients (COP), and medication management and use (MMU)—and self‐reported nursing performance. Analyses included confirmatory factor analysis (CFA) with multigroup measurement invariance testing across hospital types, multiple regression (variance inflation factor [VIF] = 3.07–5.69; Durbin–Watson = 1.84), relative weights analysis to address multicollinearity, the marker‐variable technique to assess common method bias (CMB), and extensive sensitivity analyses.

**Results:**

JCI implementation was moderately high, with the highest mean score for ACC (mean = 4.95 ± 1.38) and the lowest for IPSGs (mean = 4.46 ± 1.48). Self‐reported nursing performance was moderate (mean = 4.64 ± 1.39). The standards explained 67.2% of the variance in self‐reported performance (*R* ^2^ = 0.672, *p* < 0.001), with MMU (*β* = 0.277), IPSGs (*β* = 0.208), and PCC (*β* = 0.137) emerging as the strongest correlates of self‐reported nursing performance, although the cross‐sectional same‐source design precludes causal inference. ACC, AOP, and COP did not reach statistical significance. Measurement invariance testing achieved full scalar invariance (ΔCFI = 0.008), validating direct mean comparisons across hospital types. Nurses in private hospitals reported significantly higher levels of JCI standard implementation than nurses in public hospitals across all six domains (*p* < 0.001; Cohen’s *d* = −0.60 to −0.89). The common method variance (CMV)–adjusted *R* ^2^ was 0.59.

**Conclusion:**

JCI patient‐centered standards showed significant positive associations with self‐reported nursing performance in Yemen. Given the cross‐sectional, same‐source self‐report design, these findings should be interpreted as correlational rather than causal, even after common‐method‐bias adjustment (CMV‐adjusted *R* ^2^ = 0.59). Targeted training in medication safety and patient safety protocols, coupled with equitable resource allocation and systemic investment in public hospitals, is recommended to close quality gaps and improve outcomes for vulnerable Yemeni populations.

**Implications for Nursing Management:**

Nurse leaders should treat medication safety, IPSG‐anchored supervision, and patient‐centered communication as priority levers for quality improvement. They should also advocate for equitable resourcing and structural support of public‐sector nursing to close the implementation gap with private hospitals.

## 1. Introduction

Healthcare quality is fundamental to effective medical services, driving societal progress, and improving the quality of life [1, 2]. The World Health Organization (WHO) underscores the importance of universal health coverage (UHC), particularly in low‐ and middle‐income countries (LMICs) [3]. In Yemen, prolonged conflict and economic instability have severely strained the healthcare system, leading to resource shortages, staffing deficits, and infrastructure deterioration [4]. These challenges have compromised care delivery, heightened the risk of medical errors, and undermined patient safety, necessitating the establishment of a standardized framework for quality of care.

Nurses, who are pivotal to healthcare delivery, face significant barriers in Yemen, including inadequate training, heavy workload, and limited resources [5]. For instance, Al‐Jaradi et al. [6] reported that only 16.8% of nurses in public hospitals in Sana′a had a good level of knowledge of drug administration, with 64.2% at a fair level, highlighting the gaps in medication management skills. A 2021 ministry of Health assessment revealed that only 5% of public and private hospitals achieved high‐quality benchmarks, underscoring systemic gaps [7]. These deficiencies, coupled with the low caring efficacy among nurses (mean = 3.99) reported by Al‐Thaifani et al. [8], point to the need for urgent interventions to enhance nursing practice and elevate the standards of care in the country.

The Joint Commission International (JCI) provides a globally recognized framework of patient‐centered standards designed to improve healthcare quality and safety through accreditation [9]. These standards encompass key domains, such as patient safety, access to care, and patient rights. Previous studies have demonstrated their benefits in several contexts [10, 11]. In South Korea, nurses in JCI‐accredited hospitals reported improved patient safety, although gaps in the implementation of International Patient Safety Goals (IPSGs) persist [10]. In China, JCI standards have been associated with improved nursing management and enhanced patient identification and hand hygiene, thereby increasing patient satisfaction [11]. Medication management standards, such as those for titrations, have raised concerns about care delays and documentation burden [12], whereas cultural and leadership barriers hinder their adoption in diverse settings [13]. In the Middle East, JCI standards have been associated with better care quality and positive nursing perceptions; however, their application in resource‐scarce settings, such as Yemen, remains underexplored. Insights from Guatemala highlight the difficulties of implementing JCI standards in low‐income contexts, where infrastructure and staffing constraints pose barriers, illustrating the importance of tailored strategies, such as staff training and leadership commitment [14, 15].

For nurse leaders, alignment between accreditation standards and bedside nursing practice is a strategic priority. When JCI patient‐centered standards are coherently embedded in unit workflows, charge nurses, nurse managers, and directors of nursing gain a shared language for setting priorities, allocating staff, structuring training, and monitoring quality. Conversely, weak alignment fragments accountability: leaders are left navigating between accreditation expectations and the reality of resource constraints, which can erode psychological safety, increase moral distress, and undermine retention—particularly in fragile, conflict‐affected systems such as Yemen, where nurse leaders are often the de facto custodians of safety culture.

Examining the relationship between JCI patient‐centered standards and nurses’ self‐reported performance directly informs nursing leadership decisions about which standards to prioritize for in‐service training, supervisory coaching, and resource investment, especially when leaders must do more with less. This study addresses this knowledge gap.

Resource constraints and inconsistent standardization have hampered nursing performance in Yemeni hospitals, increasing the risk of medical errors and compromising patient care. This study addresses this gap by examining the implementation of JCI patient‐centered standards and their association with self‐reported nursing performance in Sana′a, Yemen, a setting with severe resource limitations. We hypothesized that JCI standards are positively associated with self‐reported nursing performance. By assessing adherence to key standards, including IPSG, access to care and continuity (ACC), and medication management and use (MMU), and their relationship with performance effectiveness, efficiency, and indicators, this research aims to provide actionable recommendations for improving healthcare quality in resource‐constrained environments.

## 2. Materials and Methods

### 2.1. Study Design

This cross‐sectional study examined the association between the implementation of JCI patient‐centered standards and self‐reported nursing performance in hospitals in Sana′a, Yemen, from August to October 2024. A cross‐sectional design was selected because of its suitability for studying social phenomena, such as the implementation of JCI standards and their association with self‐reported nursing performance, allowing a snapshot of current practices across multiple hospitals. The study was approved by the Ethics Committee of the Center of Business Administration, Sana′a University (approval no. 835, dated July 15, 2024) to ensure compliance with ethical standards. All participants provided written informed consent prior to participation, and confidentiality was ensured through anonymous data collection. The study was conducted in compliance with the ethical principles outlined in the Declaration of Helsinki, which ensures the protection of the rights, safety, and well‐being of participants. Institutional permission was obtained from each participating hospital prior to data collection, and participation was entirely voluntary, with no coercion.

This study was reported in accordance with the Strengthening the Reporting of Observational Studies in Epidemiology (STROBE) guidelines for cross‐sectional studies (see Supporting File S4).

### 2.2. Setting

Data were collected from August to October 2024 across six hospitals in Sana′a, Yemen: Al‐Thawra General Hospital (public, 550 beds, not JCI‐accredited), 48 Model Hospital (public, 360 beds, not JCI‐accredited), Azal Model Hospital (private, 110 beds, not JCI‐accredited), Modern European Hospital (private, 90 beds, not JCI‐accredited), Royal Hospital (private, 70 beds, not JCI‐accredited), and Yemeni Health International Hospital (private, 30 beds, not JCI‐accredited). These urban facilities varied in size and provided a diverse sample for assessing the implementation of JCI standards. None of the participating hospitals had JCI accreditation at the time of data collection.

### 2.3. Participants and Sample Size

Of the 1459 nursing personnel listed on human resources rosters across the six participating hospitals, 847 were licensed registered nurses (RNs) or licensed practical nurses (LPNs) actively employed in emergency departments, inpatient wards, intensive care units (ICUs), or neonatal units who met the inclusion criteria. The inclusion criterion was willingness to participate, and the exclusion criteria were nurses in outpatient or helper roles, those not working in the specified departments, and those with incomplete data. Using simple random sampling with proportional representation across hospitals, we distributed 710 questionnaires to account for anticipated nonresponse and to ensure adequate statistical power for the planned analyses. A total of 621 questionnaires were returned, yielding a response rate of 87.5%. After excluding 95 cases (59 with incomplete data, 24 in outpatient or helper roles, and 12 not employed in the specified departments), the final analytic sample comprised 526 nurses (304 from public hospitals, 57.8%; 222 from private hospitals, 42.2%).

**FIGURE 1:**
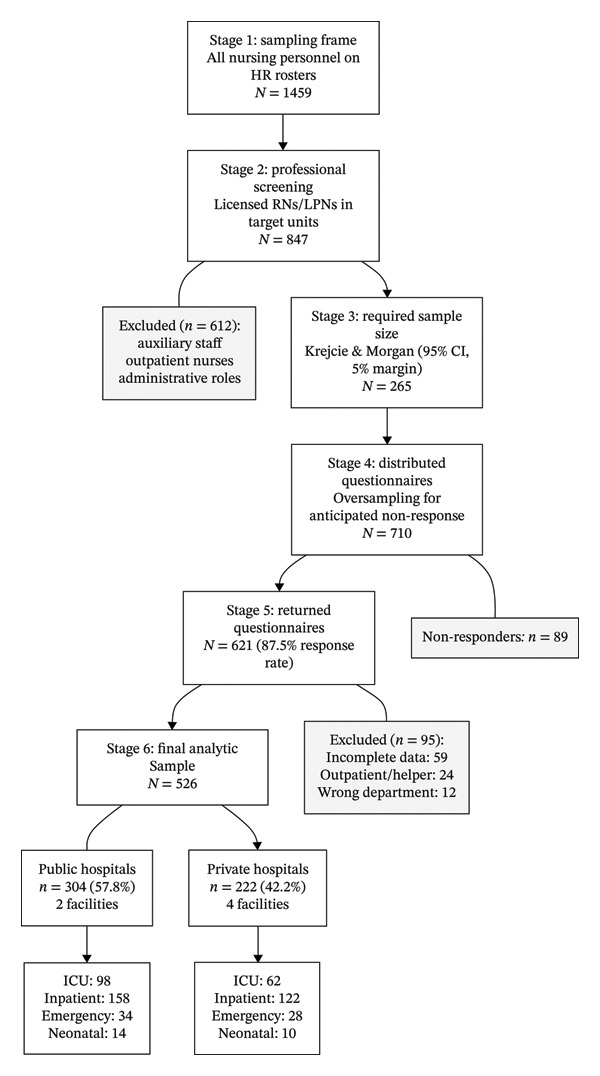
STROBE‐compliant participant flow diagram. Stage 1: Sampling frame (*N* = 1459) of all nursing personnel on HR rosters. Stage 2: Professional screening (*N* = 847) of licensed RNs/LPNs in target units (ICU, inpatient, emergency, and neonatal). Stage 3: Required sample size (*N* = 265) using Krejcie and Morgan (95% CI, 5% margin). Stage 4: Distributed questionnaires (*N* = 710) with 27% oversampling for anticipated nonresponse. Stage 5: Returned questionnaires (*N* = 621; 87.5% response rate). Stage 6: Final analytic sample (*N* = 526) after excluding incomplete data (*n* = 59), outpatient/helper roles (*n* = 24), and nurses from nontarget departments (*n* = 12). The final sample comprised 304 public‐hospital and 222 private‐hospital nurses. Abbreviations: HR, human resources; ICU, intensive care unit; LPN, licensed practical nurse; RN, registered nurse; STROBE, Strengthening the Reporting of Observational Studies in Epidemiology.

The minimum required sample size for this study was 265, calculated using the Krejcie and Morgan formula [16] for a finite population of 847 nurses (95% confidence level and 5% margin of error). To ensure robust statistical power (> 99%) for the complex structural equation modelling (SEM) and multigroup confirmatory factor analyses (CFAs) performed in this study, we substantially oversampled and distributed 710 questionnaires. A post‐hoc power analysis confirmed that the achieved sample size was adequate. For the multiple regression model with six predictors and an observed *R* ^2^ of 0.672, the achieved power exceeded 0.999. For the independent *t*‐tests comparing hospital types with Cohen’s *d* of 0.5, the achieved power exceeded 0.999. The participant selection process is illustrated in Figure 1.

### 2.4. Variables

The independent variable was the implementation level of JCI patient‐centered standards, comprising six domains: IPSGs, ACC, patient‐centered care (PCC), AOP, care of patients (COP), and MMU. The dependent variable was self‐reported nursing performance, measured across three dimensions: performance effectiveness, performance efficiency, and performance indicators. Hospital type (public vs. private) and years of experience were included as covariates in the analyses.

### 2.5. Data Sources and Measurement

Data were collected using a two‐part questionnaire developed for this study and administered in Arabic language. The complete questionnaire, including the item‐to‐construct mapping matrix and verbatim English wording of all items, is provided in Supporting File S4 (Tables S1.1–S1.4). The questionnaire targeted nurses’ perspectives on field practices, given their extensive involvement in implementing JCI standards and their practical knowledge compared with other healthcare or administrative staff. It comprised 91 items.

Part 1 (25 items) collected demographic data, including hospital name, department, job title, and years of experience.

Part 2 (66 items) assessed patient‐centered standards (42 items) covering six domains—IPSGs (9 items), ACC (7 items), PCC (7 items), AOP (5 items), COP (8 items), and MMU (6 items)—adapted from JCI (2024) and previous studies [17–20]; and self‐reported nursing performance (24 items) assessing three dimensions—performance effectiveness (4 items), performance efficiency (5 items), and performance indicators (15 items)—adapted from Schwirian [21], the National Database of Nursing Quality Indicators (NDNQI), and previous studies [21–24]. Responses were recorded on a 7‐point Likert scale (1 = strongly disagree to 7 = strongly agree).

Composite scores were constructed as the mean of all items within each domain. Missing item responses within scales were handled using mean imputation for scales with ≤ 20% missing items; otherwise, the cases were excluded from the analysis. Higher values indicate stronger implementation and higher self‐reported performance. No item was reverse‐coded.

The questionnaire was developed by synthesizing these sources and validated using a two‐step process. First, a panel of four experts reviewed the questionnaire for content validity (item‐level content validity index [I‐CVI] = 0.75–1.00; scale‐level CVI based on the average method [S‐CVI/Ave] = 0.92). We acknowledge that the four‐member expert panel is below the commonly recommended 5–10 experts for content validation. This constraint is revisited in the Limitations section and should be considered when interpreting the instrument’s evidentiary base. Second, a pilot test with 40 nurses from two hospitals in Sana′a (excluded from the final sample) confirmed the clarity and cultural appropriateness of the instrument. The average completion time was approximately 20–25 min.

Psychometric properties were evaluated using CFA in IBM SPSS AMOS. Both the six‐factor JCI standards model and the three‐factor self‐reported nursing performance model were validated, with fit indices meeting acceptable thresholds (root mean square error of approximation [RMSEA] ≤ 0.08; comparative fit index [CFI] ≥ 0.90; Supporting File S2, Table S2.3) and factor loadings between 0.41 and 0.88. The CFA path diagram for the six‐factor JCI standards model is presented in Supporting File S2, Figure S2.1. Convergent validity was confirmed, with composite reliability (CR) of 0.885–0.932 and average variance extracted (AVE) of 0.513–0.749. Reliability was high, with Cronbach’s *α* = 0.979 for the JCI standards, 0.952 for self‐reported nursing performance, and 0.982 overall.

### 2.6. Bias

Selection bias was minimized through simple random sampling with proportional representation across hospitals. Nonresponse bias was reduced by distributing 710 questionnaires, which achieved an overall response rate of 87.5%. Information bias was mitigated by using a standardized, pretested questionnaire with anonymous data collection to reduce social desirability bias. Incomplete responses were excluded to ensure the quality of the data. Potential confounders were addressed by stratifying the sample by hospital type and department and controlling for them in the statistical analyses.

Common method bias (CMB) was assessed using Harman’s single‐factor test. The unrotated factor analysis revealed that the first factor explained 43.06% of the variance, which was below the 50% threshold. To further address CMB, we applied the marker‐variable technique [25] using nurses’ years of service as the marker variable to statistically adjust the key correlations and *R* ^2^ estimates.

### 2.7. Study Hypotheses

This study aimed to test the following hypotheses:

 H1: The implementation of JCI patient‐centered standards is significantly associated with self‐reported nursing performance in Yemeni hospitals.
 H2: There is a significant difference in the implementation of JCI standards between public and private hospitals.

### 2.8. Statistical Methods

Data were analyzed using IBM SPSS Statistics (version 26.0) and IBM SPSS AMOS. Descriptive statistics were used to summarize JCI implementation and self‐reported nursing performance. Normality was confirmed via skewness and kurtosis (within the ±2 threshold), justifying the use of parametric tests. Psychometric properties (Cronbach’s *α*, CR, and AVE), CFA model fit indices, and correlation matrices are detailed in Supporting File S2 (Tables S2.1–S2.4).

Measurement invariance across hospital types (configural, metric, and scalar) was assessed using multigroup CFA in AMOS, relying on the change in CFI (ΔCFI) recommended by Chen [26], where ΔCFI ≤ 0.010 indicates invariance. Owing to the known hypersensitivity of the *χ* ^2^ difference test in large samples, invariance decisions were based on the ΔCFI criterion rather than the traditional *χ* ^2^ difference test [27].

Multiple regression was performed (VIF = 3.07–5.69; Durbin–Watson = 1.84). Because moderate multicollinearity could bias individual coefficients, we additionally conducted a relative weights analysis [28] to provide stable estimates of each standard’s contribution.

To address CMB, the marker‐variable technique [25] was applied using nurses’ years of service as a theoretically unrelated marker variable. This technique adjusts the observed correlations and *R* ^2^ estimates to provide a more conservative estimate of the true associations.

Hypotheses were tested using (1) simple linear regression (overall association, reporting *R* ^2^ and 95% CI), (2) multiple linear regression (individual JCI contributions, reporting standardized *β*, *p* values, and 95% CI), and (3) independent *t*‐tests with Bonferroni correction (*α* = 0.0083 for six comparisons) and Cohen’s *d* effect sizes. The complete regression output and collinearity diagnostics are presented in Supporting File S3 (Table S3.1).

Model diagnostics included residual plots, Cook’s distance, and the Breusch–Pagan test for heteroscedasticity. The regression diagnostic plots are provided in Supporting File S3 (Figure S3.1). Missing data were handled using listwise deletion. Sensitivity analyses comprised stratified regressions (by hospital type and department), exclusion of influential observations (Cook’s *D* > 4/*n*), factor score regression, and multilevel modelling.

## 3. Results

### 3.1. Participants

The final sample consisted of 526 nurses from six hospitals in Sana′a, with an 87.5% response rate. The majority of participants (*n* = 304, 57.8%) were from public hospitals, and the remainder (*n* = 222, 42.2%) were from private hospitals (Table 1).

**TABLE 1:** Study population and sample distribution across hospitals in Sana′a, Yemen (*N* = 526).

Hospital	Study population (*N*)	Distributed (*n*)	Returned (*n*)	Analyzed (*n*)	Analyzed (%)
Al‐Thawra General (public)	320	230	196	170	32.3
48 Model (public)	224	170	152	134	25.5
Azal Model (private)	150	130	120	103	19.6
Modern European (private)	70	75	65	51	9.7
Royal (private)	50	70	55	40	7.6
Yemeni Health International (private)	33	35	33	28	5.3
Total	847	710	621	526	100.0

*Note:* Response rate = returned/distributed = 621/710 = 87.5%. Final sample distribution: public, 304 (57.8%); private, 222 (42.2%). No statistical tests were applied to this table.

### 3.2. Characteristics of Study Participants

The demographic characteristics of the 526 participants are presented in Table 2. The sample was drawn from emergency, inpatient, intensive care, and neonatal units. Most participants worked in general wards (53.2%, *n* = 280), followed by the ICU (30.4%, *n* = 160). The sample had a small female majority (52.3%, *n* = 275), consistent with the nursing workforce in Yemen. The largest group of participants had 3–6 years of experience (37.8%, *n* = 199). Regarding qualifications, 54.8% (*n* = 288) held diplomas and 43.5% (*n* = 229) held bachelor’s degrees.

**TABLE 2:** Demographic characteristics of nursing staff in Sana′a, Yemen (*N* = 526).

Variable	Category	Frequency (*n*)	Percentage (%)
Hospital type	Public	304	57.8
	Private	222	42.2

Department	ICU	160	30.4
	General ward	280	53.2
	Emergency	62	11.8
	Neonatal unit	24	4.6

Sex	Male	251	47.7
	Female	275	52.3

Experience	< 3 years	116	22.1
	3–6 years	199	37.8
	7–10 years	91	17.3
	> 10 years	120	22.8

Qualification	Diploma	288	54.8
	Bachelor’s	229	43.5
	Higher studies	9	1.7

*Note:* No statistical tests were performed. “Higher studies” included master’s degrees or above. Abbreviations: ICU, Intensive Care Unit.

### 3.3. Implementation Level of Patient‐Centered JCI Standards

As shown in Table 3, the overall implementation of the JCI patient‐centered standards was rated moderately high (mean = 4.77 ± 1.43). Among the six domains, ACC received the highest score (mean = 4.95 ± 1.38), reflecting robust practices of timely and coordinated care. In contrast, IPSGs scored the lowest (mean = 4.46 ± 1.48), indicating a weaker adherence to safety protocols.

**TABLE 3:** Implementation levels of JCI patient‐centered standards among nurses in Sana′a, Yemen (*N* = 526).

Standard	Mean	SD
ACC	4.95	1.381
COP	4.88	1.389
PCC	4.85	1.376
MMU	4.76	1.464
AOP	4.69	1.471
IPSG	4.46	1.482
Overall	4.77	1.427

*Note:* Means and standard deviations were derived from a 7‐point Likert scale (1 = strongly disagree, 7 = strongly agree). No inferential tests were conducted. Standards are ordered from highest to lowest mean.

Abbreviations: ACC, Access to Care and Continuity; AOP, Assessment of Patients; COP, Care of Patients; IPSG, International Patient Safety Goals; MMU, Medication Management and Use; PCC, Patient‐Centered Care.

### 3.4. Self‐Reported Nursing Performance Levels

The average self‐reported nursing performance was moderate (mean = 4.64 ± 1.39). Performance effectiveness was the highest (mean = 4.86 ± 1.32), followed by performance efficiency (mean = 4.65 ± 1.45), and performance indicators scored the lowest (mean = 4.41 ± 1.40) (Table 4).

**TABLE 4:** Self‐reported nursing performance levels among nurses in Sana′a, Yemen (*N* = 526).

Dimension	Mean	SD
Performance Effectiveness	4.86	1.318
Performance Efficiency	4.65	1.448
Performance Indicators	4.41	1.401
**Overall**	**4.64**	**1.389**

*Note:* Means and standard deviations were derived from a 7‐point Likert scale (1 = strongly disagree, 7 = strongly agree). No inferential tests were conducted. Dimensions are ordered from the highest to the lowest mean.

### 3.5. Testing the Study Hypotheses

Data normality was confirmed for all key variables, as the skewness and kurtosis values were within the acceptable threshold of ±2. For example, the IPSG had a skewness of −0.428 and kurtosis of −0.426. This finding supports the use of parametric tests for hypothesis testing (see Table 5).

**TABLE 5:** Normality test results for the key study variables (*N* = 526).

Variable	Mean	SD	Skewness	Kurtosis
JCI overall	4.77	1.427	−0.553	−0.278
ACC	4.95	1.381	−0.525	0.017
COP	4.88	1.389	−0.502	−0.049
PCC	4.85	1.376	−0.564	0.095
MMU	4.76	1.464	−0.507	−0.127
AOP	4.69	1.471	−0.437	−0.265
IPSG	4.46	1.482	−0.428	−0.426
Nursing performance	4.64	1.389	−0.552	0.265

*Note:* Skewness and kurtosis values within ±2 indicate a normal distribution, justifying the use of parametric tests.

Abbreviations: ACC, Access to Care and Continuity; AOP, Assessment of Patients; COP, Care of Patients; IPSG, International Patient Safety Goal; JCI, Joint Commission International; MMU, Medication Management and Use; PCC, Patient‐Centered Care.

Simple linear regression revealed that the overall implementation of JCI standards was significantly associated with self‐reported nursing performance (*R* = 0.820, *R* ^2^ = 0.672, adjusted *R* ^2^ = 0.668; *p* < 0.001), explaining 67.2% of the variance in performance. Multiple regression analysis was conducted to identify the contributions of individual JCI standards. The model identified MMU (*β* = 0.277, *p* < 0.001), IPSGs (*β* = 0.208, *p* < 0.001), and PCC (*β* = 0.137, *p* = 0.003) as significant positive correlates of self‐reported nursing performance. ACC (*β* = −0.057, *p* = 0.160), AOP (*β* = 0.076, *p* = 0.070), and COP (*β* = 0.090, *p* = 0.083) were not statistically significant at *α* = 0.05. Table 6 presents these results.

**TABLE 6:** Regression analysis of the association between JCI standards and self‐reported nursing performance (*N* = 526).

Model/Predictor	*β*	*p*	95% CI
Overall model			
JCI standards	—	< 0.001	*R* ^2^ = 0.672, Adj. *R* ^2^ = 0.668, *F* = 176.99
Multiple regression			
MMU	0.277	< 0.001	[0.21, 0.35]
IPSG	0.208	< 0.001	[0.14, 0.28]
PCC	0.137	0.003	[0.05, 0.23]
AOP	0.076	0.070	[−0.01, 0.16]
COP	0.090	0.083	[−0.01, 0.19]
ACC	−0.057	0.160	[−0.14, 0.02]

*Note: β* = standardized regression coefficient; CI = confidence interval. VIF range = 3.07–5.69; Durbin–Watson = 1.84; Breusch–Pagan *p* = .937. The overall simple regression *R* ^2^ indicates the total variance explained by all JCI standards combined. Predictors are ordered by descending *β* magnitude in the multiple regression.

Abbreviations: ACC, Access to Care and Continuity; AOP, Assessment of Patients; COP, Care of Patients; IPSG, International Patient Safety Goal; JCI, Joint Commission International; MMU, Medication Management and Use; PCC, Patient‐Centered Care; VIF, Variance Inflation Factor.

### 3.6. Common Method Bias and Relative Weights Analysis

To address concerns about CMB, the marker‐variable technique was applied using nurses’ years of service as the marker variable. The unadjusted *R* ^2^ value was 0.672. After adjusting for the estimated method bias, the common method variance (CMV)‐adjusted *R* ^2^ remained robust at approximately 0.59, indicating that the association between JCI standards and self‐reported nursing performance was not solely attributable to CMV.

A relative weights analysis was conducted to decompose the total *R* ^2^ (0.672) and determine the proportional contribution of each domain. This analysis confirmed that MMU was the largest contributor, accounting for approximately 23.0% of the explained variance, followed by IPSGs at 18.8%, COP at 16.4%, AOP at 15.5%, PCC at 15.3%, and ACC at 10.9%. These results demonstrate that all six domains contributed meaningfully to the prediction of self‐reported nursing performance, even though three domains did not reach statistical significance in the standard multiple regression analysis owing to multicollinearity.

### 3.7. Measurement Invariance Testing

Measurement invariance across hospital types was assessed using a multigroup CFA in AMOS. Owing to the hypersensitivity of the *χ* ^2^ test in large samples (*N* = 526), invariance was evaluated using the ΔCFI criterion. The results strongly support the measurement invariance (Table 7).

**TABLE 7:** Measurement invariance testing across hospital types (AMOS; *N* = 526).

Model	*χ* ^2^	df	CFI	RMSEA	Δ*χ* ^2^	Δdf	ΔCFI	Invariance
Configural	4002.98	1608	0.864	0.053	—	—	—	Baseline
Metric	4067.18	1644	0.862	—	64.20	36	0.002	Supported
Scalar	4253.03	1686	0.854	—	185.85	42	0.008	Supported

*Note:* Owing to the hypersensitivity of the *χ* ^2^ test in large samples (*N* = 526), invariance was evaluated using the ΔCFI criterion (Chen, 2007). A ΔCFI ≤ 0.010 indicates supported invariance, confirming both metric and full scalar invariance; ΔCFI, change in comparative fit index.

Abbreviations: CFI, Comparative Fit Index; RMSEA, Root Mean Square Error of Approximation.

The configural model showed an acceptable fit: *χ* ^2^ = 4002.98, df = 1608, CFI = 0.864, RMSEA = 0.053. The metric model (equal factor loadings) yielded CFI = 0.862, with ΔCFI = 0.002, supporting metric invariance. Most importantly, the scalar model (equal intercepts) yielded CFI = 0.854, with ΔCFI = 0.008, well below the 0.010 threshold. Because full scalar invariance was supported, the public–private differences reflected genuine differences in reported implementation rather than differential item functioning (DIF) across groups. These results confirmed that direct mean comparisons between public‐ and private‐hospital nurses were valid. A multigroup CFA model illustrating configural, metric, and scalar invariance is presented in Supporting File S2, Figure S2.2.

### 3.8. Differences in JCI Standards by Hospital Type

Independent‐samples *t*‐tests with Bonferroni correction (*α* = 0.0083 for six comparisons) were conducted to compare the implementation of JCI standards between private (*n* = 222) and public (*n* = 304) hospitals. As shown in Table 8, nurses in private hospitals reported significantly higher levels of JCI standard implementation than nurses in public hospitals in all six domains (*p* < 0.001). Effect sizes ranged from medium (ACC: *d* = −0.60) to large (IPSG: *d* = −0.89), with private hospitals consistently demonstrating higher implementation scores than public hospitals. The mean differences were substantial, ranging from 0.65 for ACC (5.33 vs. 4.68) to 0.96 for IPSG (5.01 vs. 4.05). Because scalar invariance was supported (ΔCFI = 0.008), these differences reflect true systemic disparities in reported implementation rather than DIF.

**TABLE 8:** Differences in JCI standard implementation by hospital type (*N* = 526).

Standard	Hospital type	*n*	Mean	SD	*t*	*p*	Cohen’s d
IPSG	Private	222	5.01	0.921	10.422	< 0.001	−0.89
	Public	304	4.05	1.173			

AOP	Private	222	5.19	0.978	8.622	< 0.001	−0.73
	Public	304	4.32	1.325			

MMU	Private	222	5.20	0.982	7.409	< 0.001	−0.62
	Public	304	4.45	1.345			

PCC	Private	222	5.28	0.974	7.829	< 0.001	−0.67
	Public	304	4.54	1.179			

COP	Private	222	5.29	0.905	7.690	< 0.001	−0.65
	Public	304	4.58	1.208			

ACC	Private	222	5.33	0.968	6.970	< 0.001	−0.60
	Public	304	4.68	1.181			

*Note:* Independent‐samples *t*‐tests with Bonferroni correction (*α* = 0.0083). A negative Cohen’s *d* indicates higher scores for private hospitals. Because scalar invariance was supported (ΔCFI = 0.008), these differences reflect true group disparities.

Abbreviations: ACC, Access to Care and Continuity; AOP, Assessment of Patients; COP, Care of Patients; IPSG, International Patient Safety Goal; MMU, Medication Management and Use; PCC, Patient‐Centered Care.

### 3.9. Sensitivity Analyses

Sensitivity analyses confirmed the robustness of the main findings (Supporting File S3; Figure S3.2). Stratified regression by hospital type yielded significant models for both public (*R* ^2^ = 0.642) and private (*R* ^2^ = 0.618) hospitals, although the assessment of patients predicted performance only in private facilities (*β* = 0.174, *p* = 0.010). After excluding 35 influential observations (Cook’s distance > 4/*n*), the model fit improved (*R* ^2^ = 0.718), and ACC and assessment of patients became significant. Factor score regression (*R* ^2^ = 0.636) confirmed these findings while eliminating multicollinearity concerns. Multilevel modelling revealed minimal hospital‐level clustering (intraclass correlation coefficient [ICC] = 0.022), with fixed effects consistent with single‐level results. Department‐stratified analyses showed comparable fits for the ICU (*R* ^2^ = 0.658) and inpatient units (*R* ^2^ = 0.702) (Table 9).

**TABLE 9:** Sensitivity analysis summary.

Analysis	*n*	*R* ^2^	Adj. *R* ^2^	*F* *p* value
Full sample	526	0.672	0.668	< 0.001
Public only	304	0.642	0.634	< 0.001
Private only	222	0.618	0.607	< 0.001
Excluding influential observations	491	0.718	0.714	< 0.001
ICU only	160	0.658	0.645	< 0.001
Inpatient only	280	0.702	0.696	< 0.001

*Note:* All models were significant at *p* < 0.001. “Excluding influential observations” refers to the model after removing observations with Cook’s distance > 4/*n*.

Abbreviation: ICU, Intensive Care Unit.

### 3.10. Structural Equation Model (AMOS) Results

As a robustness check, a structural equation model (SEM) was estimated using IBM SPSS AMOS to examine the overall relationship between JCI standards and self‐reported nursing performance. The model showed an acceptable fit (Supporting File S2, Figure S2.4). The overall JCI standards construct was strongly associated with self‐reported nursing performance (*β* = 0.885, *p* < 0.001), explaining 78.4% of the variance (*R* ^2^ = 0.784). The individual path coefficients from the six JCI domains to self‐reported nursing performance were as follows: IPSG (*β* = 0.334, *p* < 0.001), ACC (*β* = 0.031, *p* = 0.426, not significant), PCC (*β* = 0.118, *p* = 0.003), AOP (*β* = 0.174, *p* < 0.001), COP (*β* = 0.228, *p* < 0.001), and MMU (*β* = 0.601, *p* < 0.001). These findings confirm the main regression results and provide additional evidence of the robustness of the association between JCI standards and self‐reported nursing performance. The standardized direct, indirect, and total effects for all paths are detailed in Supporting File S2, Table S2.5. The direct path model with individual domain effects is illustrated in Supporting File S2, Figure S2.4.

## 4. Discussion

This study provides crucial insights into the implementation of JCI patient‐centered standards and their association with self‐reported nursing performance within the fragile and resource‐constrained healthcare system in Sana′a, Yemen. The findings confirm that JCI standards are positively associated with self‐reported nursing performance. However, their implementation is uneven, and profound disparities exist between public and private facilities, reflecting the systemic challenges documented in similar LMICs, particularly those affected by conflict.

The implementation of JCI standards was moderately high, suggesting a general commitment to quality improvement in the participating hospitals. However, the particularly low score for IPSGs is a critical concern. This finding aligns with regional studies reporting deficits in nurses’ knowledge of safety protocols [6, 29]; however, it must be understood within the broader context of Yemen’s healthcare landscape. The Yemeni health system has been described as being in a state of near collapse, with only half of its health facilities fully functional because of the prolonged conflict [4]. In such an environment, the consistent application of safety protocols is severely hampered not only by knowledge gaps but also by a chronic lack of essential resources, from basic sanitation supplies to functional equipment, such as personal protective equipment (PPE). Therefore, a low IPSG score is likely to indicate a health system that struggles to maintain even the most fundamental functions of care. This suggests that deficits in patient safety are a global challenge in resource‐limited settings and are exacerbated by systemic failures rather than individual shortcomings [30, 31].

Similarly, the finding that overall self‐reported nursing performance was moderate—with performance effectiveness rated highest—points to nurses’ adaptive strategies. In severely under‐resourced and understaffed settings, healthcare professionals are often forced to prioritize immediate, life‐sustaining tasks over systematic, process‐oriented duties that underpin broader quality metrics [32]. The lower score on performance indicators, which depend on consistent documentation and tracking, further supports this interpretation, as resource constraints and shared responsibilities may hinder the measurement of outcomes. This pattern reflects the reality that final patient outcomes are not the sole responsibility of the nursing staff but are shared with nursing leadership and hospital administration, in addition to patient‐related factors. Despite its significant association with performance, the moderate score for PCC also reflects the challenges of implementing patient‐centered approaches in a strained environment, aligning with local findings of low caring efficacy among nurses [8].

Despite these challenges, our study confirmed that JCI standards are strongly associated with self‐reported nursing performance, explaining nearly two‐thirds of the variance (*R* ^2^ = 0.672). The CMV‐adjusted *R* ^2^ of 0.59 confirms that this association remains substantial even after accounting for CMB. MMU, IPSGs, and PCC were the standards most strongly associated with self‐reported nursing performance. Because all variables were measured concurrently from a single source, these relationships are best understood as covariational patterns rather than as causal effects, even after the marker‐variable analysis and the CMV‐adjusted estimate of *R* ^2^ = 0.59 are taken into account. Confirmatory tests in longitudinal or multisource designs remain necessary before firm causal claims can be advanced. The strong associations of MMU, IPSG, and PCC align with findings from more stable healthcare systems. For example, studies in China and South Korea have demonstrated that implementing JCI patient safety goals is associated with measurable improvements in patient identification, staff communication, and overall safety culture [10, 11]. The fact that these core standards retained their predictive power in a challenging setting such as Yemen reinforces their universal importance as foundational pillars of safe nursing care.

Conversely, the nonsignificant associations of ACC, AOP, and COP in the standard multiple regression analysis warrant careful consideration. These nonsignificant associations are highly likely to be mathematical artifacts of the high intercorrelations among the JCI subscales (VIFs ≈ 5.6) rather than a true absence of association. The relative weights analysis confirmed that all six domains contributed meaningfully to the prediction of self‐reported nursing performance: MMU (23.0%), IPSG (18.8%), COP (16.4%), AOP (15.5%), PCC (15.3%), and ACC (10.9%). These standards are highly system‐dependent and require a robust infrastructure for patient tracking, interdepartmental communication, and care planning. While nurses demonstrate commitment to these principles, in a fragmented health system with critical health worker shortages and disrupted referral pathways, the infrastructure needed to support these standards is largely lacking. Therefore, their lack of statistical significance in the standard multiple regression is less of an indictment of the standards themselves than a reflection of multicollinearity, which suppresses individual coefficients.

The most striking finding was the substantially higher reported level of JCI standard implementation in private hospitals compared to that in public hospitals. We framed this difference as one of self‐reported implementation rather than a direct comparison of clinical performance, since no objective performance indicators were measured. Nevertheless, the magnitude of these differences (Cohen’s *d* = −0.60 to −0.89) suggests an implementation gap that nurse leaders, hospital administrators, and policymakers should address to advance the equity of care. This is consistent with a well‐documented phenomenon in LMICs, where the private sector often demonstrates greater responsiveness and resource availability [33]. In Yemen, private facilities primarily serve higher‐income populations and can invest in resources and training to meet accreditation standards. This stands in stark contrast to public hospitals, which serve the majority of the population but are crippled by conflict, irregular salary payments, severe supply shortages, and damaged infrastructure, as noted by the Yemeni Ministry of Health [7]. This disparity creates a two‐tiered system of reported implementation, with potential consequences for equity of care, highlighting the critical issue of health equity, in which the most vulnerable populations receive care in facilities that are least equipped to ensure quality and safety.

Measurement invariance testing provides crucial context for interpreting hospital‐type comparisons in this study. The achievement of full scalar invariance (ΔCFI = 0.008) indicates that both factor loadings and item intercepts are equivalent across public and private hospitals. This means that nurses from public and private hospitals interpreted the questionnaire items in the same way; therefore, the observed mean differences reflect true systemic differences in implementation levels rather than DIF or measurement artifacts. We can therefore confidently state that higher scores in private hospitals reflect true systemic differences in reported implementation (resource‐driven) and not DIF.

### 4.1. Implications for Nursing Leadership and Management

The findings have several implications for nurse leaders and managers. First, the standards most strongly associated with self‐reported performance—MMU, IPSGs, and PCC—offer concrete, leader‐actionable levers: structured medication‐safety rounds, IPSG‐anchored bedside huddles, and patient‐centered communication training are feasible even in resource‐constrained settings. Second, the substantially higher reported implementation in private hospitals signals an equity challenge that is, in part, a leadership and governance challenge: chief nursing officers and unit managers in public hospitals require explicit mandates, protected time, and budget authority to translate accreditation standards into practice. Third, the moderate self‐reported performance scores reinforce the importance of shared accountability: frontline nurses cannot be expected to compensate for systemic gaps in supplies, staffing, and infrastructure, and nurse leaders should advocate upward for the structural conditions that make standard‐aligned care possible. Finally, the lower reported implementation of IPSGs—the most safety‐critical domain—should be treated by nursing leadership as a priority signal for targeted supervision, mentoring, and just‐culture reporting systems.

These findings have implications for both policy and practice. Although targeted training on high‐impact standards, such as MMU and IPSG, is essential and has proven effective in the local context [19, 34], such interventions are insufficient on their own. They must be coupled with systemic investment to achieve meaningful and sustainable improvements in nursing performance, particularly in the public sector. Policymakers and international partners must prioritize equitable resource allocation, health‐workforce support, and the strengthening of health‐system governance. Without addressing these fundamental challenges, the gap between standards and practice will persist, and the full benefits of quality improvement initiatives, such as the JCI, will remain out of reach for most Yemenis.

## 5. Limitations

This study has several important limitations that should be carefully considered when interpreting the findings.

First, the cross‐sectional design precludes causal inferences; observed associations may reflect reverse causation (nurses with higher self‐reported performance may report better JCI implementation) or omitted variable bias (unmeasured factors, such as management quality, may influence both constructs). Longitudinal research is required to establish temporal precedence in this regard.

Second, the dependent variable was conceptualized as self‐reported nursing performance, which is conceptually fragile and vulnerable to social desirability bias. Without objective indicators (e.g., medication error rates, supervisor ratings, or patient outcomes), the performance construct relies entirely on nurses’ subjective self‐assessments, which may not accurately reflect true clinical performance.

Third, CMB remains a significant concern. All key variables were collected simultaneously from the same respondents using identical 7‐point Likert scales, which may have inflated the observed associations. Although Harman’s single‐factor test (43.06%) fell below the 50% threshold, it is widely recognized as an insufficient diagnostic. Our marker‐variable adjustment [25] reduced the *R* ^2^ from 0.672 to approximately 0.59, confirming that the variance explained, while still substantial, was partially inflated by method bias. Readers should interpret the magnitude of these associations with caution.

Fourth, content validity was established with a panel of four experts (two hospital administrators and two nursing researchers), which is below the commonly recommended 5–10 experts. Although the I‐CVI values (0.75–1.00) and the S‐CVI/Ave (0.92) met conventional thresholds, a larger and more diverse panel—including frontline nurse leaders, clinical educators, and quality officers—would have strengthened the content validity evidence and broadened the representation of clinical, educational, and leadership perspectives. Findings derived from this instrument should therefore be interpreted with this constraint in mind, and replication studies should employ expanded expert panels.

Fifth, moderate multicollinearity among the JCI subscales (VIF = 3.07–5.69; correlations *r* = 0.62–0.93) complicated the interpretation of individual regression coefficients. The relative weights analysis confirmed that all domains contributed meaningfully; however, the standard multiple regression coefficients for ACC, AOP, and COP should be interpreted with caution. A sensitivity analysis that removed influential observations revealed that ACC and AOP became significant, suggesting that these findings are less robust than those for MMU, IPSGs, and PCC.

Sixth, multilevel modelling revealed minimal hospital‐level clustering (ICC = 0.022); however, the small number of hospitals (*n* = 6) limited the statistical power to detect meaningful between‐hospital variation. With only six clusters, the precision of the variance‐component estimates was low, and the generalizability of hospital‐level inferences was severely limited.

Seventh, the generalizability of the findings is limited to urban hospital nurses in Sana′a; these findings cannot be extrapolated to rural healthcare settings, other regions of Yemen, or other healthcare professionals. The sample included only six urban hospitals and only nurses, excluding other healthcare professionals and rural settings. Replication in diverse contexts is required to establish the external validity of these findings.

## 6. Conclusion

This study demonstrates that JCI patient‐centered standards are significantly associated with self‐reported nursing performance in Yemen’s resource‐constrained hospitals, with MMU, IPSGs, and PCC identified as the standards most consistently associated with higher self‐reported performance. These cross‐sectional, same‐source associations should not be interpreted causally. However, uneven implementation—particularly lower safety scores and profound public–private disparities—threatens the quality and equity of care. Full scalar invariance (ΔCFI = 0.008) supports valid direct mean comparisons across hospital types, confirming that the observed differences reflect true systemic disparities in reported implementation rather than measurement artifacts. The large variance explained (*R* ^2^ = 0.672) is likely inflated by the CMB inherent in the same‐source self‐report design; the CMV‐adjusted estimate of *R* ^2^ = 0.59 provides a more conservative benchmark. The generalizability of this study is restricted to hospitals in urban Sana′a. To improve outcomes, policymakers should prioritize targeted training in medication safety and patient safety protocols, equitable resource allocation, and systemic strengthening of public hospitals.

For nursing leadership and management, these findings suggest a clear agenda: prioritize medication safety and IPSG‐focused supervision, embed PCC in everyday workflows, and advocate for the equitable resourcing of public‐sector nursing. In fragile health systems, nurse leaders are pivotal mediators between accreditation expectations and the realities of bedside care, and their structural empowerment is essential to sustaining quality and equity.

## Author Contributions

Kamal Ahmed Qabban conceptualized the study, designed the questionnaire, supervised data collection, and drafted the manuscript. Muneer Musleh Al‐Wesabi contributed to the study design and performed statistical analyses. Haitham Mohammed Jowah drafted the manuscript and reviewed it for important intellectual content.

## Funding

This study did not receive any specific grants from funding agencies in the public, commercial, or not‐for‐profit sectors.

## Disclosure

An earlier version of this article was posted on the Research Square preprint server on May 7, 2025 [35]. All the authors have read and approved the final manuscript.

## Ethics Statement

This study was approved by the Ethics Committee of the Center of Business Administration, Sana′a University on July 15, 2024 (approval no. 835). All participants provided written informed consent prior to participation, and confidentiality was ensured through anonymous data collection. The study was conducted in compliance with the ethical principles outlined in the Declaration of Helsinki, which ensures the protection of the rights, safety, and well‐being of participants. Institutional permissions were obtained from each participating hospital prior to data collection, and participation was entirely voluntary, with no coercion.

## Consent

Consent is not required because no individual participant data (e.g., images or identifiable information) were included in this study.

## Conflicts of Interest

The authors declare no conflicts of interest.

## Supporting Information

Additional supporting information can be found online in the Supporting Information section.

## Supporting information


**Supporting Information** The following supporting information is available online: Supporting File S1: Study questionnaire and item‐to‐construct mapping. This file provides the complete two‐part questionnaire used for data collection. Part 1 included 25 demographic and situational questions. Part 2 included 66 scored items assessing JCI patient‐centered standards (42 items across 6 domains) and self‐reported nursing performance (24 items across 3 dimensions), all rated on a 7‐point Likert scale. Table S1.1 presents the complete item‐to‐construct mapping matrix; Table S1.2 presents the 25 demographic items; and Tables S1.3 and S1.4 present the complete list of 66 scored items with verbatim English wording. Supporting File S2: Psychometric properties, CFA, measurement invariance, and SEM. This file contains the detailed validation of the measurement instruments and the SEM results, including the following: Table S2.1 (psychometric properties: Cronbach’s *α*, CR, and AVE); Table S2.2 (CFA model fit indices and factor loadings for the JCI patient‐centered standards [six‐factor model, 42 items]); Table S2.3 (CFA model fit indices and factor loadings for the nursing performance model [three‐factor model, 24 items]); Table S2.4 (correlation matrix among JCI patient‐centered standards and nursing performance); Table S2.5 (standardized direct, indirect, and total effects from the SEM); Figure S2.1 (CFA path diagram for the six‐factor JCI patient‐centered standards model); Figure S2.2 (multigroup CFA measurement invariance across public and private hospitals); Figure S2.3 (CFA path diagram for the three‐factor self‐reported nursing performance model); and Figure S2.4 (SEM path diagram showing the second‐order structural model). Supporting File S3: Regression diagnostics, complete regression results, common‐method variance diagnostics, relative weights analysis, and sensitivity analyses. This file contains the following sections: Section A, complete multiple regression results with collinearity diagnostics (Table S3.1); Section B, regression diagnostic plots (Figure S3.1); Section C, relative weights analysis of JCI domains predicting nursing performance (Table S3.2); Section D, common method variance (CMV) diagnostics, including Harman’s single‐factor test, common latent factor analysis, and the marker‐variable technique with CMV‐adjusted estimates (Table S3.3); and Section E, summary sensitivity analyses comprising a forest plot of JCI subscale scores by hospital type, Cohen’s *d* effect sizes, standardized regression coefficients with 95% confidence intervals, and model *R* ^2^ comparisons (Figure S3.2). Supporting File S4: STROBE Checklist for cross‐sectional studies.

## Data Availability

The datasets generated and analyzed in this study are not publicly available because of ethical restrictions protecting participants’ confidentiality. However, they are available from the corresponding author (Kamal Ahmed Qabban; q.qubban.cba@su.edu.ye) upon reasonable request and subject to approval by the Ethics Committee of Sana′a University. A de‐identified dataset, codebook, and analysis syntax are available upon reasonable request.
