# Supplementary material for: Association Between Joint Commission International Patient‐Centered Standards and Self‐Reported Nursing Performance in Sana′a, Yemen Hospitals
Source: J Nurs Manag. 2026 May 30;2026:8353270. doi: 10.1155/jonm/8353270 (PMC13239347; doi:10.1155/jonm/8353270)

## Slide 1
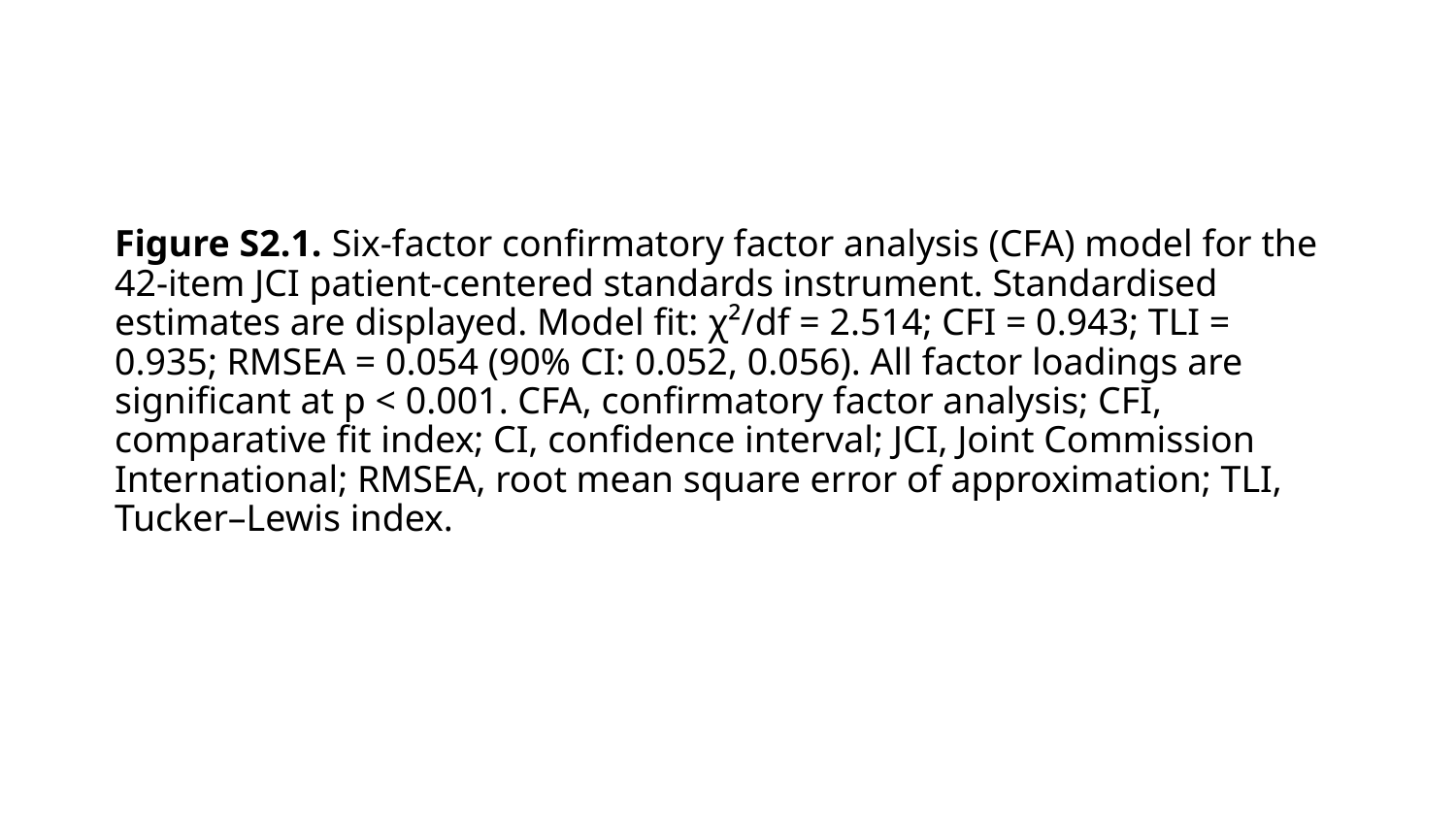

Figure S2.1. Six-factor confirmatory factor analysis (CFA) model for the 42-item JCI patient-centered standards instrument. Standardised estimates are displayed. Model fit: χ²/df = 2.514; CFI = 0.943; TLI = 0.935; RMSEA = 0.054 (90% CI: 0.052, 0.056). All factor loadings are significant at p < 0.001. CFA, confirmatory factor analysis; CFI, comparative fit index; CI, confidence interval; JCI, Joint Commission International; RMSEA, root mean square error of approximation; TLI, Tucker–Lewis index.

## Slide 2
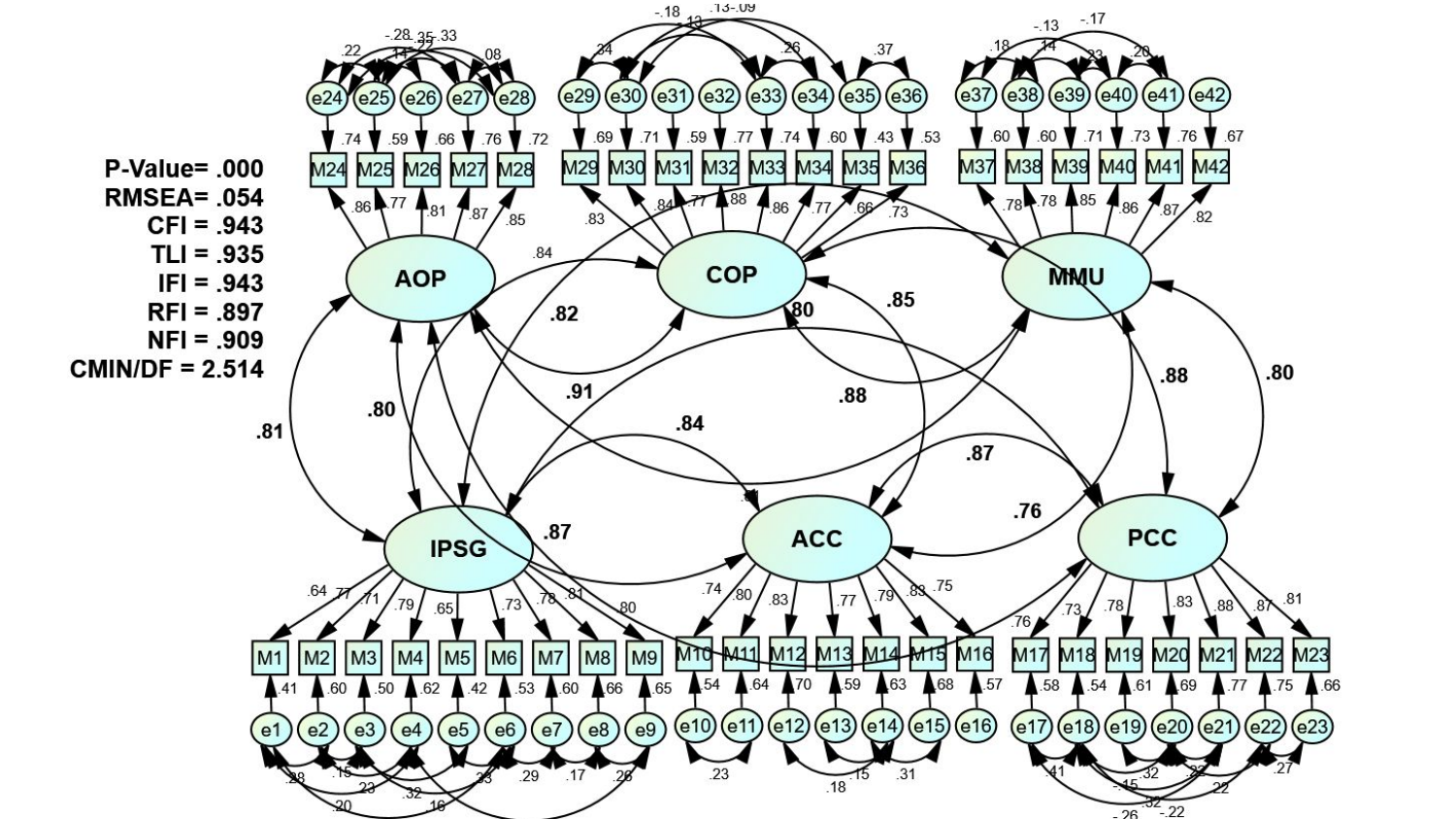

Supplement: Supplementary file 1 — Supporting Information The following supporting information is available online: Supporting File S1: Study questionnaire and item‐to‐construct mapping. This file provides the complete two‐part questionnaire used for data collection. Part 1 included 25 demographic and situational questions. Part 2 included 66 scored items assessing JCI patient‐centered standards (42 items across 6 domains) and self‐reported nursing performance (24 items across 3 dimensions), all rated on a 7‐point Likert scale. Table S1.1 presents the complete item‐to‐construct mapping matrix; Table S1.2 presents the 25 demographic items; and Tables S1.3 and S1.4 present the complete list of 66 scored items with verbatim English wording. Supporting File S2: Psychometric properties, CFA, measurement invariance, and SEM. This file contains the detailed validation of the measurement instruments and the SEM results, including the following: Table S2.1 (psychometric properties: Cronbach’s α, CR, and AVE); Table S2.2 (CFA model fit indices and factor loadings for the JCI patient‐centered standards [six‐factor model, 42 items]); Table S2.3 (CFA model fit indices and factor loadings for the nursing performance model [three‐factor model, 24 items]); Table S2.4 (correlation matrix among JCI patient‐centered standards and nursing performance); Table S2.5 (standardized direct, indirect, and total effects from the SEM); Figure S2.1 (CFA path diagram for the six‐factor JCI patient‐centered standards model); Figure S2.2 (multigroup CFA measurement invariance across public and private hospitals); Figure S2.3 (CFA path diagram for the three‐factor self‐reported nursing performance model); and Figure S2.4 (SEM path diagram showing the second‐order structural model). Supporting File S3: Regression diagnostics, complete regression results, common‐method variance diagnostics, relative weights analysis, and sensitivity analyses. This file contains the following sections: Section A, complete multiple regression results wit [file JONM-2026-8353270-s001.zip › S2_1_Figure.pptx]
