# Supplementary material for: Association Between Joint Commission International Patient‐Centered Standards and Self‐Reported Nursing Performance in Sana′a, Yemen Hospitals
Source: J Nurs Manag. 2026 May 30;2026:8353270. doi: 10.1155/jonm/8353270 (PMC13239347; doi:10.1155/jonm/8353270)
Supplement: Supplementary file 1 — Supporting Information The following supporting information is available online: Supporting File S1: Study questionnaire and item‐to‐construct mapping. This file provides the complete two‐part questionnaire used for data collection. Part 1 included 25 demographic and situational questions. Part 2 included 66 scored items assessing JCI patient‐centered standards (42 items across 6 domains) and self‐reported nursing performance (24 items across 3 dimensions), all rated on a 7‐point Likert scale. Table S1.1 presents the complete item‐to‐construct mapping matrix; Table S1.2 presents the 25 demographic items; and Tables S1.3 and S1.4 present the complete list of 66 scored items with verbatim English wording. Supporting File S2: Psychometric properties, CFA, measurement invariance, and SEM. This file contains the detailed validation of the measurement instruments and the SEM results, including the following: Table S2.1 (psychometric properties: Cronbach’s α, CR, and AVE); Table S2.2 (CFA model fit indices and factor loadings for the JCI patient‐centered standards [six‐factor model, 42 items]); Table S2.3 (CFA model fit indices and factor loadings for the nursing performance model [three‐factor model, 24 items]); Table S2.4 (correlation matrix among JCI patient‐centered standards and nursing performance); Table S2.5 (standardized direct, indirect, and total effects from the SEM); Figure S2.1 (CFA path diagram for the six‐factor JCI patient‐centered standards model); Figure S2.2 (multigroup CFA measurement invariance across public and private hospitals); Figure S2.3 (CFA path diagram for the three‐factor self‐reported nursing performance model); and Figure S2.4 (SEM path diagram showing the second‐order structural model). Supporting File S3: Regression diagnostics, complete regression results, common‐method variance diagnostics, relative weights analysis, and sensitivity analyses. This file contains the following sections: Section A, complete multiple regression results wit [file JONM-2026-8353270-s001.zip › Supplementary_File_S1_R3.docx]

# Supplementary File S1: Study Questionnaire and Item-to-Construct Mapping

The study questionnaire was developed as a two-part instrument administered in Arabic. Part 1 includes 25 demographic questions covering hospital name, department, job title, years of experience, sex, age, nationality, educational level, qualification, training, and other background variables. Part 2 includes 66 scored items measured on a 7-point Likert scale (1 = strongly disagree to 7 = strongly agree): 42 items assessing six Joint Commission International (JCI) patient-centered standards domains—International Patient Safety Goals (IPSG), Access to Care and Continuity (ACC), Patient-Centered Care (PCC), Assessment of Patients (AOP), Care of Patients (COP), and Medication Management and Use (MMU)—and 24 items assessing three self-reported nursing performance dimensions (Performance Effectiveness, Performance Efficiency, and Performance Indicators).

A complete item-to-construct mapping matrix was developed and validated by an expert panel prior to data collection. The matrix ensures that each questionnaire item is explicitly linked to its respective construct and sub-dimension. Content validity was established using the Content Validity Index (CVI) method, with item-level CVI (I-CVI) values ranging from 0.75 to 1.00 and a scale-level CVI based on the average method (S-CVI/Ave) of 0.92, both exceeding the recommended thresholds.

## Table S1.1. Item-to-construct mapping for the 66-item scored questionnaire.

| **Construct** | **Sub-dimension/Domain** | **Item numbers** | **No. of items** |
| --- | --- | --- | --- |
| JCI patient-centered standards | International Patient Safety Goals (IPSG) | IPSG1–IPSG9 | 9 |
|  | Access to Care and Continuity (ACC) | ACC1–ACC7 | 7 |
|  | Patient-Centered Care (PCC) | PCC1–PCC7 | 7 |
|  | Assessment of Patients (AOP) | AOP1–AOP5 | 5 |
|  | Care of Patients (COP) | COP1–COP8 | 8 |
|  | Medication Management and Use (MMU) | MMU1–MMU6 | 6 |
|  | Total JCI items |  | 42 |
| Self-reported nursing performance | Performance Effectiveness (PFE) | PFE1–PFE4 | 4 |
|  | Performance Efficiency (PFI) | PFI1–PFI5 | 5 |
|  | Performance Indicators (PIND) | PIND1–PIND15 | 15 |
|  | Total performance items |  | 24 |
| Total scored items |  |  | 66 |

*Note. JCI, Joint Commission International.*

## Complete Questionnaire Items

### Part 1: Demographic questions (25 items)

**Table S1.2. Demographic and background questionnaire items (Part 1).**

| **No.** | **Item description** | **Response options/format** |
| --- | --- | --- |
| 1 | Hospital type | Government ( ); Private ( ) |
| 2 | Hospital name | _____________________ |
| 3 | Sex | Male ( ); Female ( ) |
| 4 | Age | _____________________ |
| 5 | Educational qualification | Diploma ( ); Bachelor's ( ); Master's ( ); Other ( ) |
| 6 | Specialty | _____________________ |
| 7 | Graduation year | _____________________ |
| 8 | Years of service | _____________________ |
| 9 | Shift type | Day ( ); Night ( ); Rotating ( ) |
| 10 | Job title | Staff nurse ( ); Head nurse ( ); Other ( ) |
| 11 | Department | Emergency ( ); Inpatient ward ( ); ICU ( ); Neonatal unit ( ); Other ( ) |
| 12 | Patient load (average per shift) | _____________________ |
| 13 | Year of employment at current hospital | _____________________ |
| 14 | Do you have another job? | Yes ( ); No ( ) |
| 15 | Marital status | Single ( ); Married ( ); Divorced ( ); Widowed ( ) |
| 16 | Have you received training on JCI standards? | Yes ( ); No ( ) |
| 17 | If yes, specify the type of training | _____________________ |
| 18 | Years of experience in current department | _____________________ |
| 19 | Have you participated in quality improvement projects? | Yes ( ); No ( ) |
| 20 | Average working hours per week | _____________________ |
| 21 | Do you work overtime regularly? | Yes ( ); No ( ) |
| 22 | Have you received patient safety training? | Yes ( ); No ( ) |
| 23 | Are you a member of a professional nursing organisation? | Yes ( ); No ( ) |
| 24 | Have you experienced a patient safety incident in the past year? | Yes ( ); No ( ) |
| 25 | If yes, describe briefly | _____________________ |

*Note. These 25 items were not scored on the Likert scale and were used for descriptive and stratification purposes only. ICU, intensive care unit; JCI, Joint Commission International.*

### Part 2A: JCI patient-centered standards (42 items)

**Table S1.3. JCI patient-centered standards items (42 items).**

| **Item code** | **Item (verbatim English)** |
| --- | --- |
| International Patient Safety Goals (IPSG) – 9 items | International Patient Safety Goals (IPSG) – 9 items |
| IPSG1 | The hospital develops and implements a process to improve the accuracy of patient identification. |
| IPSG2 | The hospital develops and implements a process to improve the effectiveness of communication among caregivers. |
| IPSG3 | The hospital develops and implements a process for the safe use of high-alert medications. |
| IPSG4 | The hospital develops and implements a process to ensure correct-site, correct-procedure, correct-patient surgery. |
| IPSG5 | The hospital develops and implements a process to reduce the risk of healthcare-associated infections. |
| IPSG6 | The hospital develops and implements a process for the effective management of clinical alarm systems. |
| IPSG7 | The hospital develops and implements a process to reduce the risk of patient harm resulting from falls. |
| IPSG8 | The hospital develops and implements a process for the management of fire safety risks. |
| IPSG9 | The hospital develops and implements a process to reduce the risk of unintended retention of foreign objects in surgical procedures. |
| Access to Care and Continuity (ACC) – 7 items | Access to Care and Continuity (ACC) – 7 items |
| ACC1 | Patient care is continuous and coordinated among caregivers and across care settings. |
| ACC2 | The hospital designs processes to support continuity, coordination, and integration of care. |
| ACC3 | Patients and families are educated about the care process and how to continue care at home. |
| ACC4 | The hospital ensures timely access to care and appropriate placement based on clinical needs. |
| ACC5 | Transfer of patients between units or facilities is coordinated to ensure continuity of care. |
| ACC6 | Emergency care is available and accessible 24 hours a day, 7 days a week. |
| ACC7 | Patients receive care in the appropriate setting based on their clinical condition and needs. |
| Patient-Centered Care (PCC) – 7 items | Patient-Centered Care (PCC) – 7 items |
| PCC1 | Patient and family rights are respected, and care is provided in a manner that preserves dignity. |
| PCC2 | Patients and families participate in care planning and decision-making. |
| PCC3 | Cultural and spiritual values and preferences are respected and incorporated into care. |
| PCC4 | Patients and families receive adequate information about diagnosis, treatment, and prognosis. |
| PCC5 | Patient privacy and confidentiality are maintained throughout the care process. |
| PCC6 | Pain is assessed and managed appropriately for all patients. |
| PCC7 | End-of-life care is provided with compassion and respect for patient and family wishes. |
| Assessment of Patients (AOP) – 5 items | Assessment of Patients (AOP) – 5 items |
| AOP1 | All patients receive an initial assessment appropriate to their condition and care needs. |
| AOP2 | Patients are reassessed at appropriate intervals based on their condition and treatment plan. |
| AOP3 | Laboratory and diagnostic services are available and accessible to support patient assessment. |
| AOP4 | Clinical laboratory services meet quality standards and are performed by qualified personnel. |
| AOP5 | Radiological and other diagnostic services are provided safely and meet quality standards. |
| Care of Patients (COP) – 8 items | Care of Patients (COP) – 8 items |
| COP1 | Nursing care is planned, implemented, and evaluated based on patient assessment findings. |
| COP2 | Patients at high risk for specific conditions receive appropriate care and monitoring. |
| COP3 | Patient nutrition is assessed and managed to support the care plan. |
| COP4 | Patient rehabilitation needs are assessed and addressed as part of the care plan. |
| COP5 | Care is provided to minimise the risk of hospital-acquired infections. |
| COP6 | Care is provided to manage pain and other symptoms effectively. |
| COP7 | Patient care is documented accurately and comprehensively. |
| COP8 | Patient discharge planning begins early and involves the patient and family. |
| Medication Management and Use (MMU) – 6 items | Medication Management and Use (MMU) – 6 items |
| MMU1 | Medication orders are clear, complete, and reviewed for appropriateness. |
| MMU2 | Medications are prepared, dispensed, and administered safely and accurately. |
| MMU3 | Medication storage and management practices ensure safety and prevent errors. |
| MMU4 | Patients are monitored for medication effectiveness and adverse reactions. |
| MMU5 | Medication reconciliation is performed at transitions of care. |
| MMU6 | High-alert medications are managed with additional safeguards to prevent errors. |

### Part 2B: Self-reported nursing performance (24 items)

**Table S1.4. Self-reported nursing performance questionnaire items (Part 2B).**

| **Item code** | **Item (verbatim English)** |
| --- | --- |
| Performance Effectiveness (PFE) – 4 items | Performance Effectiveness (PFE) – 4 items |
| PFE1 | I complete nursing procedures efficiently and accurately within the required time frame. |
| PFE2 | I demonstrate clinical competence in providing direct patient care. |
| PFE3 | I collaborate effectively with the healthcare team to achieve optimal patient outcomes. |
| PFE4 | I maintain professional standards and ethical conduct in my nursing practice. |
| Performance Efficiency (PFI) – 5 items | Performance Efficiency (PFI) – 5 items |
| PFI1 | I prioritise patient care tasks effectively to maximise productivity. |
| PFI2 | I use time and resources efficiently in delivering patient care. |
| PFI3 | I minimise waste and unnecessary procedures while maintaining quality of care. |
| PFI4 | I adapt my work practices to accommodate changes in patient volume or acuity. |
| PFI5 | I maintain a balance between speed and quality in performing nursing duties. |
| Performance Indicators (PIND) – 15 items | Performance Indicators (PIND) – 15 items |
| PIND1 | I consistently follow infection control protocols to prevent hospital-acquired infections. |
| PIND2 | I accurately document patient care activities and clinical observations. |
| PIND3 | I effectively manage patient pain and comfort levels. |
| PIND4 | I identify and respond appropriately to changes in patient condition. |
| PIND5 | I provide patient education that supports self-care and recovery. |
| PIND6 | I ensure patient safety through adherence to safety protocols and procedures. |
| PIND7 | I communicate effectively with patients and families about care plans and procedures. |
| PIND8 | I demonstrate cultural sensitivity in my interactions with patients and families. |
| PIND9 | I participate in quality improvement initiatives to enhance patient care. |
| PIND10 | I maintain competency in emergency and critical care procedures. |
| PIND11 | I effectively manage stress and maintain resilience in challenging work situations. |
| PIND12 | I seek feedback and opportunities for professional development. |
| PIND13 | I demonstrate leadership skills in coordinating patient care activities. |
| PIND14 | I contribute to a positive work environment that supports team effectiveness. |
| PIND15 | I adhere to ethical principles and patient rights in all aspects of care. |

*Note. All items were rated on a 7-point Likert scale (1 = strongly disagree to 7 = strongly agree). No items were reverse-coded.*
