# Supplementary material for: Association Between Joint Commission International Patient‐Centered Standards and Self‐Reported Nursing Performance in Sana′a, Yemen Hospitals
Source: J Nurs Manag. 2026 May 30;2026:8353270. doi: 10.1155/jonm/8353270 (PMC13239347; doi:10.1155/jonm/8353270)
Supplement: Supplementary file 1 — Supporting Information The following supporting information is available online: Supporting File S1: Study questionnaire and item‐to‐construct mapping. This file provides the complete two‐part questionnaire used for data collection. Part 1 included 25 demographic and situational questions. Part 2 included 66 scored items assessing JCI patient‐centered standards (42 items across 6 domains) and self‐reported nursing performance (24 items across 3 dimensions), all rated on a 7‐point Likert scale. Table S1.1 presents the complete item‐to‐construct mapping matrix; Table S1.2 presents the 25 demographic items; and Tables S1.3 and S1.4 present the complete list of 66 scored items with verbatim English wording. Supporting File S2: Psychometric properties, CFA, measurement invariance, and SEM. This file contains the detailed validation of the measurement instruments and the SEM results, including the following: Table S2.1 (psychometric properties: Cronbach’s α, CR, and AVE); Table S2.2 (CFA model fit indices and factor loadings for the JCI patient‐centered standards [six‐factor model, 42 items]); Table S2.3 (CFA model fit indices and factor loadings for the nursing performance model [three‐factor model, 24 items]); Table S2.4 (correlation matrix among JCI patient‐centered standards and nursing performance); Table S2.5 (standardized direct, indirect, and total effects from the SEM); Figure S2.1 (CFA path diagram for the six‐factor JCI patient‐centered standards model); Figure S2.2 (multigroup CFA measurement invariance across public and private hospitals); Figure S2.3 (CFA path diagram for the three‐factor self‐reported nursing performance model); and Figure S2.4 (SEM path diagram showing the second‐order structural model). Supporting File S3: Regression diagnostics, complete regression results, common‐method variance diagnostics, relative weights analysis, and sensitivity analyses. This file contains the following sections: Section A, complete multiple regression results wit [file JONM-2026-8353270-s001.zip › Supplementary_File_S2_R3.docx]

# Supplementary File S2: Psychometric Properties, Confirmatory Factor Analysis, Measurement Invariance, and Structural Equation Modelling

## Table S2.1. Psychometric properties of the JCI standards and nursing performance scales.

| **Construct** | **Sub-dimension** | **Cronbach's α** | **CR** | **AVE** |
| --- | --- | --- | --- | --- |
| JCI standards | IPSG | 0.918 | 0.911 | 0.534 |
|  | ACC | 0.917 | 0.914 | 0.603 |
|  | PCC | 0.924 | 0.925 | 0.637 |
|  | AOP | 0.903 | 0.910 | 0.669 |
|  | COP | 0.926 | 0.927 | 0.616 |
|  | MMU | 0.924 | 0.922 | 0.665 |
|  | Overall JCI | 0.979 | — | — |
| Nursing performance | Performance Effectiveness | 0.869 | 0.877 | 0.588 |
|  | Performance Efficiency | 0.919 | 0.920 | 0.741 |
|  | Performance Indicators | 0.939 | 0.934 | 0.488* |
|  | Overall performance | 0.952 | — | — |

*Note. All values exceed the recommended thresholds (CR > 0.70; AVE > 0.50). *The AVE for Performance Indicators was slightly below 0.50 (0.488); however, the high composite reliability (0.934) supports the convergent validity of this dimension. ACC, Access to Care and Continuity; AOP, Assessment of Patients; AVE, average variance extracted; COP, Care of Patients; CR, composite reliability; IPSG, International Patient Safety Goals; JCI, Joint Commission International; MMU, Medication Management and Use; PCC, Patient-Centered Care.*

## Table S2.2. Standardised factor loadings for the six-factor JCI standards CFA model.

| **Domain** | **Item range** | **Factor loading range** | **Significant loadings** |
| --- | --- | --- | --- |
| IPSG | IPSG1–IPSG9 | 0.52–0.85 | 9/9 (100%) |
| ACC | ACC1–ACC7 | 0.55–0.82 | 7/7 (100%) |
| PCC | PCC1–PCC7 | 0.51–0.88 | 7/7 (100%) |
| AOP | AOP1–AOP5 | 0.61–0.86 | 5/5 (100%) |
| COP | COP1–COP8 | 0.53–0.84 | 8/8 (100%) |
| MMU | MMU1–MMU6 | 0.58–0.87 | 6/6 (100%) |

*Note. Factor loadings are from the AMOS standardised solution. All items loaded significantly (p < 0.001) on their respective factors. Model fit indices: CFI = 0.943; RMSEA = 0.054. ACC, Access to Care and Continuity; AOP, Assessment of Patients; CFA, confirmatory factor analysis; CFI, comparative fit index; COP, Care of Patients; IPSG, International Patient Safety Goals; JCI, Joint Commission International; MMU, Medication Management and Use; PCC, Patient-Centered Care; RMSEA, root mean square error of approximation.*

## Table S2.3. Standardised factor loadings for the three-factor nursing performance CFA model.

| **Dimension** | **Item range** | **Factor loading range** | **Significant loadings** |
| --- | --- | --- | --- |
| Performance Effectiveness | PFE1–PFE4 | 0.64–0.88 | 4/4 (100%) |
| Performance Efficiency | PFI1–PFI5 | 0.58–0.85 | 5/5 (100%) |
| Performance Indicators | PIND1–PIND15 | 0.41–0.86 | 15/15 (100%) |

*Note. Factor loadings are from the AMOS standardised solution. All items loaded significantly (p < 0.001) on their respective factors. Model fit indices: CFI = 0.952; RMSEA = 0.064. CFA, confirmatory factor analysis; CFI, comparative fit index; RMSEA, root mean square error of approximation.*

## Table S2.4. Pearson correlations among JCI standards domains and nursing performance (N = 526).

| **Variable** | **IPSG** | **ACC** | **PCC** | **AOP** | **COP** | **MMU** | **NP** |
| --- | --- | --- | --- | --- | --- | --- | --- |
| IPSG | 1.00 |  |  |  |  |  |  |
| ACC | 0.79* | 1.00 |  |  |  |  |  |
| PCC | 0.78* | 0.84* | 1.00 |  |  |  |  |
| AOP | 0.75* | 0.83* | 0.85* | 1.00 |  |  |  |
| COP | 0.77* | 0.84* | 0.87* | 0.88* | 1.00 |  |  |
| MMU | 0.73* | 0.80* | 0.81* | 0.82* | 0.84* | 1.00 |  |
| Nursing performance | 0.73* | 0.77* | 0.78* | 0.78* | 0.80* | 0.82* | 1.00 |

*Note. *p < 0.001; all correlations are significant. ACC, Access to Care and Continuity; AOP, Assessment of Patients; COP, Care of Patients; IPSG, International Patient Safety Goals; JCI, Joint Commission International; MMU, Medication Management and Use; NP, self-reported nursing performance; PCC, Patient-Centered Care.*

## Table S2.5. Standardised direct, indirect, and total effects from the structural equation model.

| **Path** | **Direct effect** | **Indirect effect** | **Total effect** |
| --- | --- | --- | --- |
| JCI standards → nursing performance | 0.885*** | — | 0.885*** |
| IPSG → nursing performance | 0.334*** | — | 0.334*** |
| ACC → nursing performance | 0.031 (ns) | — | 0.031 (ns) |
| PCC → nursing performance | 0.118** | — | 0.118** |
| AOP → nursing performance | 0.174*** | — | 0.174*** |
| COP → nursing performance | 0.228*** | — | 0.228*** |
| MMU → nursing performance | 0.601*** | — | 0.601*** |

*Note. Standardised coefficients are reported. ***p < 0.001; **p < 0.01; ns, not significant. The first row (JCI standards → nursing performance) is from a second-order structural model in which a higher-order JCI factor predicts the latent nursing performance composite. The remaining rows are from a separate direct path model in which the six first-order JCI domains directly predict nursing performance without a higher-order factor. Model fit for both models: χ²/df = 2.534; CFI = 0.851; RMSEA = 0.054. ACC, Access to Care and Continuity; AOP, Assessment of Patients; CFI, comparative fit index; COP, Care of Patients; IPSG, International Patient Safety Goals; JCI, Joint Commission International; MMU, Medication Management and Use; PCC, Patient-Centered Care; RMSEA, root mean square error of approximation.*

## Figures


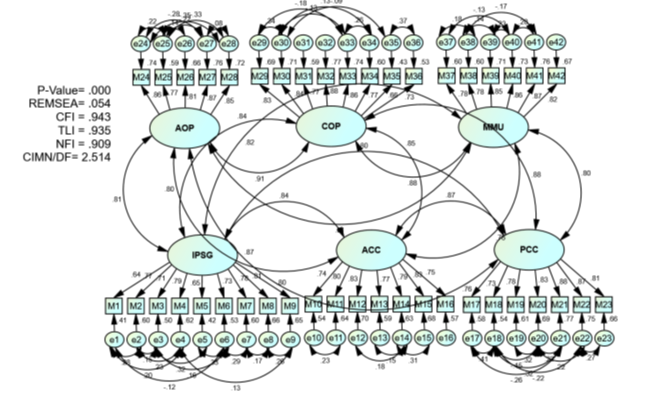


**Figure S2.1.** *Six-factor confirmatory factor analysis (CFA) model for the 42-item JCI patient-centered standards instrument. Standardised estimates are displayed. Model fit: χ²/df = 2.514; CFI = 0.943; TLI = 0.935; RMSEA = 0.054 (90% CI: 0.052, 0.056). All factor loadings are significant at p < 0.001. CFA, confirmatory factor analysis; CFI, comparative fit index; CI, confidence interval; JCI, Joint Commission International; RMSEA, root mean square error of approximation; TLI, Tucker–Lewis index.*


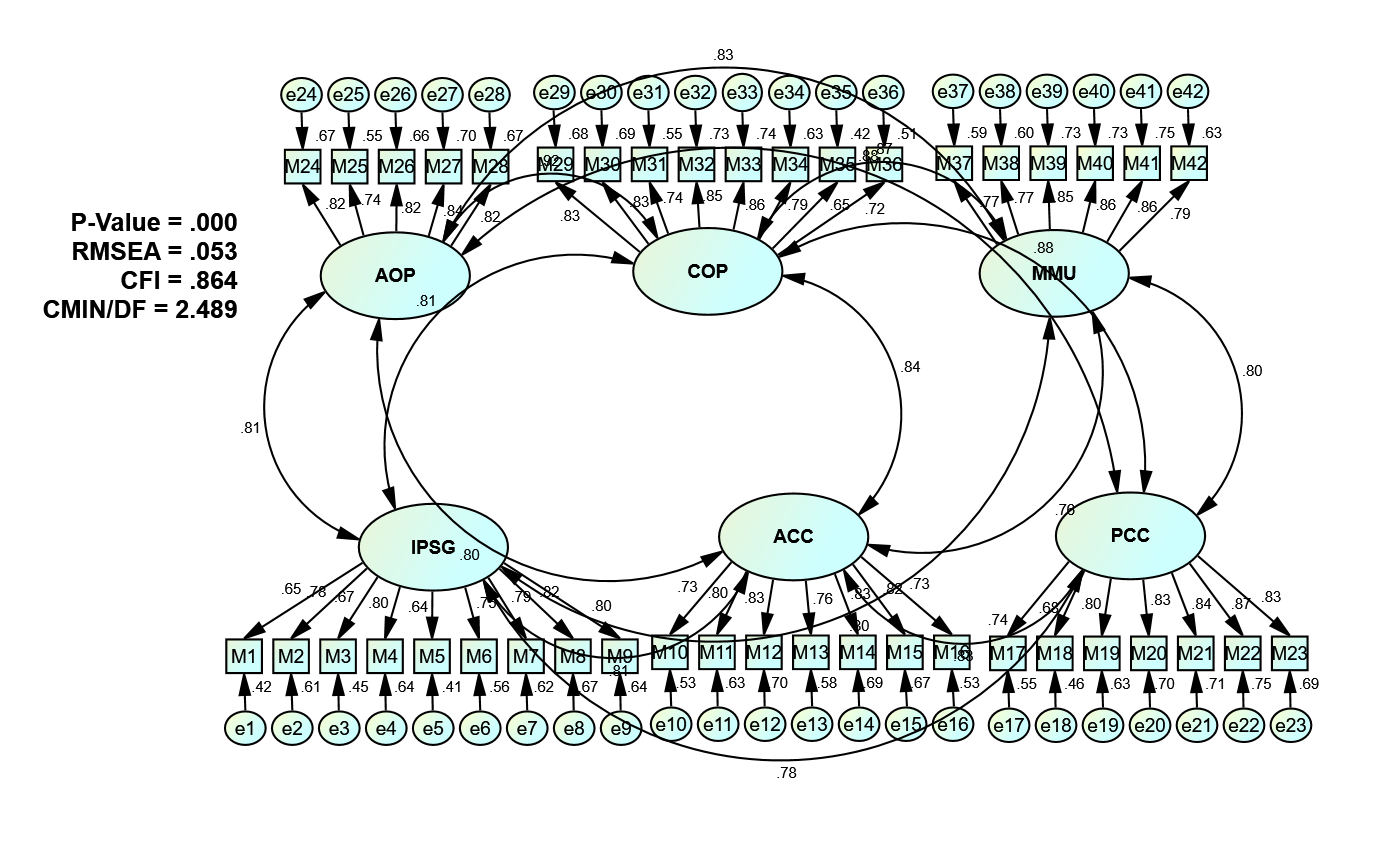


**Figure S2.2.** *Multi-group confirmatory factor analysis testing measurement invariance across public (n = 304) and private (n = 222) hospital nurses. The diagram displays the configural model (baseline). Metric invariance (ΔCFI = 0.002) and scalar invariance (ΔCFI = 0.008) were both supported. CFI, comparative fit index.*


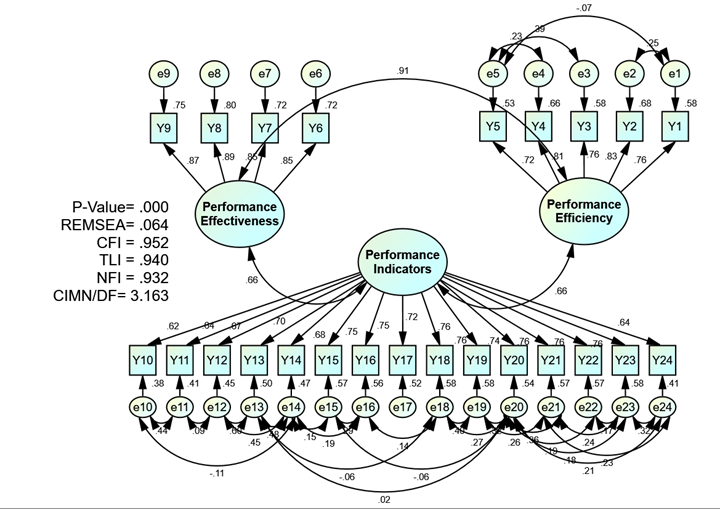


**Figure S2.3.** *Three-factor confirmatory factor analysis (CFA) model for the 24-item self-reported nursing performance instrument. Standardised estimates are displayed. Model fit: χ²/df = 3.163; CFI = 0.952; TLI = 0.940; RMSEA = 0.064 (90% CI: 0.061, 0.067). All factor loadings are significant at p < 0.001. CFA, confirmatory factor analysis; CFI, comparative fit index; CI, confidence interval; RMSEA, root mean square error of approximation; TLI, Tucker–Lewis index.*


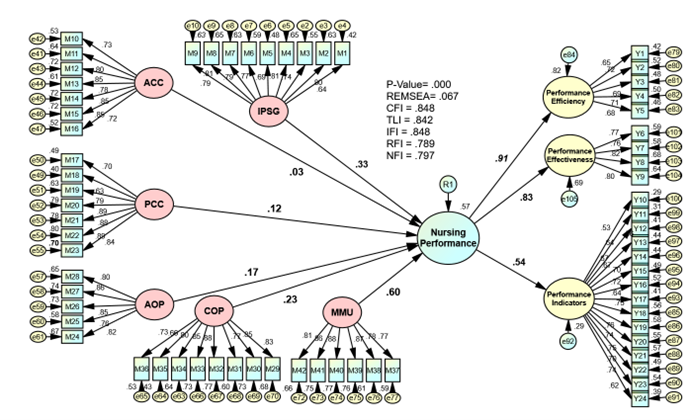


**Figure S2.4.** *Second-order structural equation model examining the association between the higher-order JCI patient-centered standards construct and self-reported nursing performance. Standardised path coefficients are displayed. The model explains 78.4% of the variance in self-reported nursing performance (R² = 0.784). Path from JCI standards to nursing performance: β = 0.885, p < 0.001. Model fit: χ²/df = 2.534; CFI = 0.851; RMSEA = 0.054. CFI, comparative fit index; JCI, Joint Commission International; RMSEA, root mean square error of approximation.*
