# Supplementary material for: Association Between Joint Commission International Patient‐Centered Standards and Self‐Reported Nursing Performance in Sana′a, Yemen Hospitals
Source: J Nurs Manag. 2026 May 30;2026:8353270. doi: 10.1155/jonm/8353270 (PMC13239347; doi:10.1155/jonm/8353270)
Supplement: Supplementary file 1 — Supporting Information The following supporting information is available online: Supporting File S1: Study questionnaire and item‐to‐construct mapping. This file provides the complete two‐part questionnaire used for data collection. Part 1 included 25 demographic and situational questions. Part 2 included 66 scored items assessing JCI patient‐centered standards (42 items across 6 domains) and self‐reported nursing performance (24 items across 3 dimensions), all rated on a 7‐point Likert scale. Table S1.1 presents the complete item‐to‐construct mapping matrix; Table S1.2 presents the 25 demographic items; and Tables S1.3 and S1.4 present the complete list of 66 scored items with verbatim English wording. Supporting File S2: Psychometric properties, CFA, measurement invariance, and SEM. This file contains the detailed validation of the measurement instruments and the SEM results, including the following: Table S2.1 (psychometric properties: Cronbach’s α, CR, and AVE); Table S2.2 (CFA model fit indices and factor loadings for the JCI patient‐centered standards [six‐factor model, 42 items]); Table S2.3 (CFA model fit indices and factor loadings for the nursing performance model [three‐factor model, 24 items]); Table S2.4 (correlation matrix among JCI patient‐centered standards and nursing performance); Table S2.5 (standardized direct, indirect, and total effects from the SEM); Figure S2.1 (CFA path diagram for the six‐factor JCI patient‐centered standards model); Figure S2.2 (multigroup CFA measurement invariance across public and private hospitals); Figure S2.3 (CFA path diagram for the three‐factor self‐reported nursing performance model); and Figure S2.4 (SEM path diagram showing the second‐order structural model). Supporting File S3: Regression diagnostics, complete regression results, common‐method variance diagnostics, relative weights analysis, and sensitivity analyses. This file contains the following sections: Section A, complete multiple regression results wit [file JONM-2026-8353270-s001.zip › Supplementary_File_S3_R3.docx]

# Supplementary File S3: Regression Diagnostics, Advanced Diagnostics, and Sensitivity Analyses

## Section A: Multiple Regression Analysis and Model Fit

This section provides the complete regression output for the multiple regression analysis predicting self-reported nursing performance from the six JCI standards domains.

### Table S3.1. Unstandardised and standardised regression coefficients with collinearity diagnostics.

| **Predictor** | **B** | **SE** | **β** | **t** | **p** | **VIF** | **Tolerance** | **95% CI** |
| --- | --- | --- | --- | --- | --- | --- | --- | --- |
| (Constant) | 0.451 | 0.206 | — | 2.189 | 0.029 | — | — | [0.05, 0.86] |
| IPSG | 0.194 | 0.035 | 0.208 | 5.543 | <0.001 | 3.070 | 0.326 | [0.13, 0.26] |
| ACC | −0.057 | 0.040 | −0.057 | −1.407 | 0.160 | 3.661 | 0.273 | [−0.14, 0.02] |
| PCC | 0.138 | 0.046 | 0.137 | 3.010 | 0.003 | 5.132 | 0.195 | [0.05, 0.23] |
| AOP | 0.072 | 0.039 | 0.076 | 1.826 | 0.070 | 3.258 | 0.307 | [−0.01, 0.16] |
| COP | 0.090 | 0.051 | 0.090 | 1.734 | 0.083 | 5.688 | 0.176 | [−0.01, 0.19] |
| MMU | 0.263 | 0.041 | 0.277 | 6.439 | <0.001 | 3.341 | 0.299 | [0.18, 0.34] |

*Note. B, unstandardised regression coefficient; β, standardised regression coefficient; SE, standard error; VIF, variance inflation factor. Durbin–Watson = 1.84; Breusch–Pagan p = 0.937 (no heteroscedasticity). ACC, Access to Care and Continuity; AOP, Assessment of Patients; CI, confidence interval; COP, Care of Patients; IPSG, International Patient Safety Goals; MMU, Medication Management and Use; PCC, Patient-Centered Care.*

## Section B: Regression Diagnostic Plots

Figure S3.1 presents the regression diagnostic plots used to evaluate the assumptions of the multiple regression model.


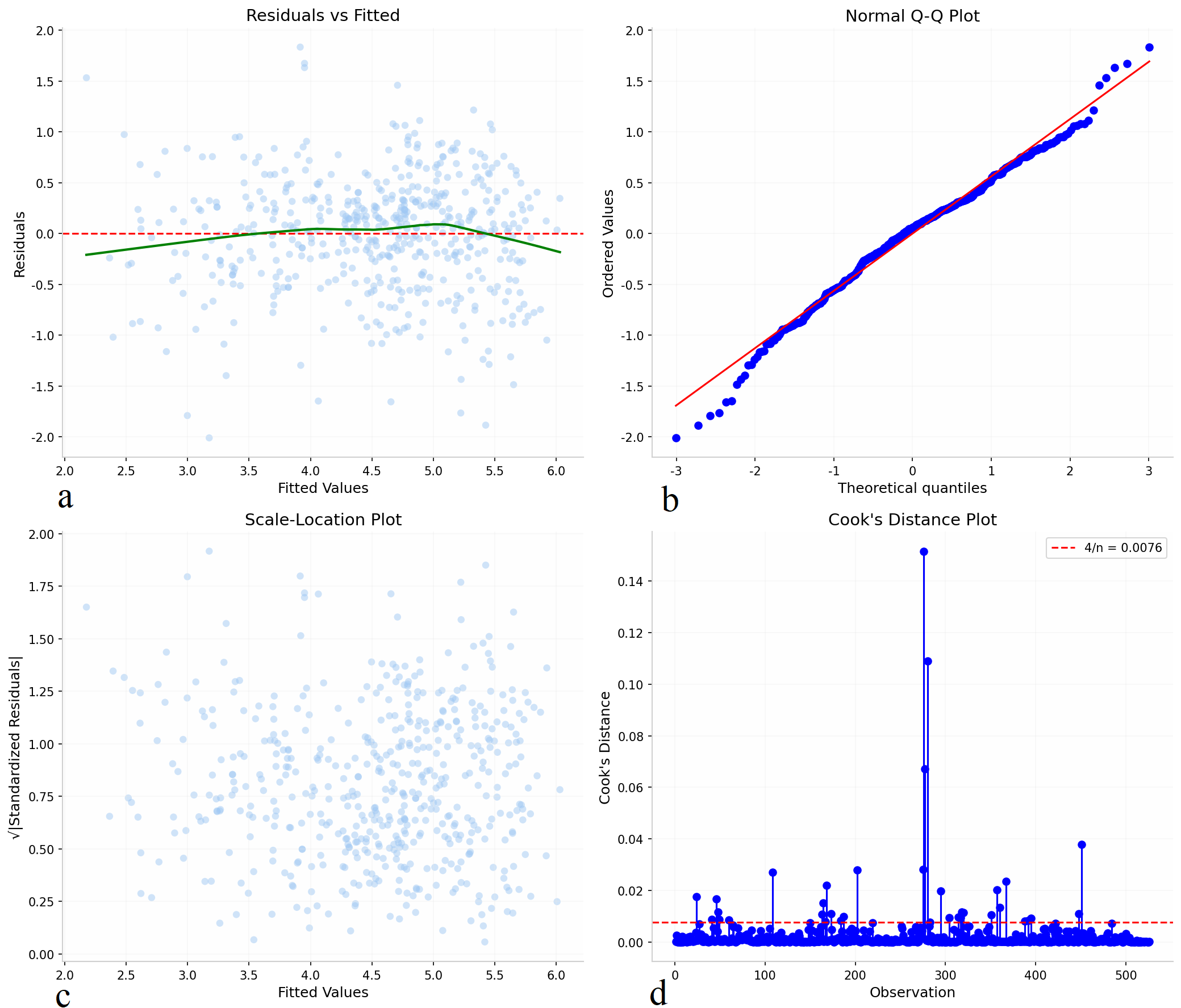


**Figure S3.1.** *Regression diagnostic plots for the multiple regression model (N = 526). The four-panel figure displays (a) residuals vs. fitted values, (b) normal Q–Q plot of standardised residuals, (c) scale–location plot, and (d) Cook's distance plot. Breusch–Pagan p = 0.937; Durbin–Watson = 1.84.*

## Section C: Relative Weights Analysis

A relative weights analysis [28] was conducted to decompose the total R² (0.672) and determine the proportional contribution of each JCI domain, accounting for multicollinearity among correlated predictors. This analysis provides a more accurate assessment of each predictor's importance than standardised regression coefficients when predictors are highly intercorrelated.

### Table S3.2. Relative weights analysis of JCI domains predicting nursing performance.

| **JCI domain** | **Relative weight** | **% of R²** | **Rank** |
| --- | --- | --- | --- |
| Medication Management and Use (MMU) | 0.155 | 23.0% | 1 |
| International Patient Safety Goals (IPSG) | 0.126 | 18.8% | 2 |
| Care of Patients (COP) | 0.110 | 16.4% | 3 |
| Assessment of Patients (AOP) | 0.104 | 15.5% | 4 |
| Patient-Centered Care (PCC) | 0.103 | 15.3% | 5 |
| Access to Care and Continuity (ACC) | 0.073 | 10.9% | 6 |
| Total | 0.672 | 100.0% |  |

*Note. Relative weights sum to the total R² (0.672). Percentages represent each domain's proportional contribution to the explained variance. The analysis was conducted using the Johnson (2000) method [28], implemented in R. JCI, Joint Commission International.*

The relative weights analysis confirmed that Medication Management and Use (MMU) was the largest contributor to the prediction of self-reported nursing performance, accounting for approximately 23.0% of the explained variance. International Patient Safety Goals (IPSG) followed at 18.8%, and Care of Patients (COP) at 16.4%. Notably, Access to Care and Continuity (ACC), which was non-significant in the standard multiple regression, still contributed 10.9% of the R², confirming that its non-significant regression coefficient was a mathematical artefact of multicollinearity rather than a true absence of association. These findings demonstrate that all six JCI domains contributed meaningfully to self-reported nursing performance.

## Section D: Advanced Common Method Variance Diagnostics

Common method variance (CMV) was assessed using three complementary techniques to provide a comprehensive evaluation of potential bias from same-source self-report data.

### Table S3.3. Common method variance diagnostic results.

| **Technique** | **Criterion** | **Result** | **Conclusion** |
| --- | --- | --- | --- |
| Harman's single-factor test | First factor < 50% | 43.06% | CMV not severe |
| Common latent factor (CLF) model | CLF R² < 25% | 43.4% shared variance | Substantial method variance present |
| Marker-variable technique [25] | Adjusted R² remains substantial | Unadjusted R² = 0.672; adjusted R² = 0.59 | Associations robust after CMV adjustment |

*Note. CLF, common latent factor; CMV, common method variance.*

Harman's single-factor test was conducted by entering all 66 items into an unrotated principal components factor analysis. The first factor explained 43.06% of the total variance, falling below the commonly cited 50% threshold. However, Harman's test is widely recognised as an insufficient and insensitive diagnostic for common method bias, as it cannot rule out the presence of CMV even when the first factor explains less than 50% of the variance.

The common latent factor (CLF) model was estimated by adding a method factor to the CFA model, with all items loading on this common factor. The CLF model indicated that approximately 43.4% of the shared variance among the items could be attributed to a common method factor. This suggests that a substantial portion of the covariance among the measured constructs may be due to shared method variance rather than true substantive relationships.

To provide a more rigorous adjustment, the marker-variable technique [25] was applied using nurses' years of service as the theoretically unrelated marker-variable. Years of service was selected because it is conceptually unrelated to both JCI implementation levels and self-reported nursing performance, yet shares the same self-report method of measurement. The smallest observed correlation between the marker-variable and the study variables was used to estimate the CMV-adjusted correlations.

The unadjusted R² for the multiple regression model was 0.672. After applying the marker-variable adjustment, the CMV-adjusted R² remained robust at approximately 0.59. This 12% reduction in explained variance (from 0.672 to 0.59) indicates that, although common method bias does inflate the observed associations, the core relationships between JCI standards and self-reported nursing performance are not solely attributable to method bias. The adjusted R² of 0.59 still represents a large effect size, confirming that the associations between JCI standards and self-reported nursing performance remain substantial even after controlling for common method variance.

*Note. The marker-variable technique provides a conservative estimate of CMV effects, assuming that the smallest observed correlation with the marker reflects pure method variance. Even after this adjustment, the same-source self-report design may continue to inflate associations to some degree. Readers should interpret the magnitude of the associations with appropriate caution.*

## Section E: Summary of Sensitivity Analyses

Figure S3.2 presents a summary of all sensitivity analyses conducted to evaluate the robustness of the main findings.


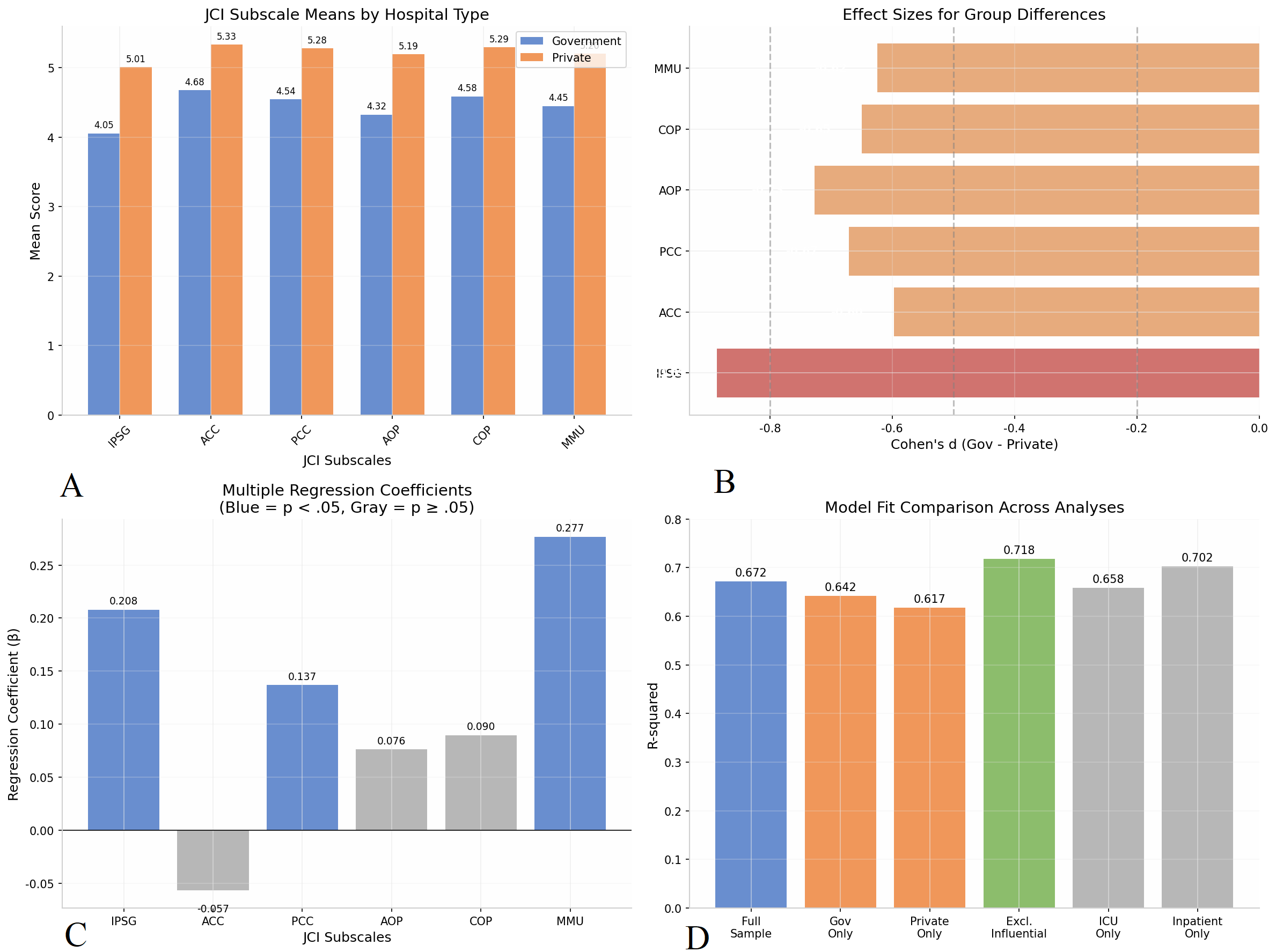


**Figure S3.2.** *Summary of sensitivity analyses. Panel A displays mean JCI subscale scores by hospital type. Panel B shows Cohen's d effect sizes for public–private differences. Panel C presents standardised regression coefficients with 95% confidence intervals. Panel D compares R² values across model specifications and subsamples. JCI, Joint Commission International.*

## References

25. Lindell MK, Whitney DJ. Accounting for common method variance in cross-sectional research designs. J Appl Psychol. 2001;86:114–21. https://doi.org/10.1037/0021-9010.86.1.114.

28. Johnson JW. A heuristic method for estimating the relative weight of predictor variables in multiple regression. Multivariate Behav Res. 2000;35:1–19. https://doi.org/10.1207/S15327906MBR3501_1.
