# Supplementary material for: Association Between Joint Commission International Patient‐Centered Standards and Self‐Reported Nursing Performance in Sana′a, Yemen Hospitals
Source: J Nurs Manag. 2026 May 30;2026:8353270. doi: 10.1155/jonm/8353270 (PMC13239347; doi:10.1155/jonm/8353270)
Supplement: Supplementary file 1 — Supporting Information The following supporting information is available online: Supporting File S1: Study questionnaire and item‐to‐construct mapping. This file provides the complete two‐part questionnaire used for data collection. Part 1 included 25 demographic and situational questions. Part 2 included 66 scored items assessing JCI patient‐centered standards (42 items across 6 domains) and self‐reported nursing performance (24 items across 3 dimensions), all rated on a 7‐point Likert scale. Table S1.1 presents the complete item‐to‐construct mapping matrix; Table S1.2 presents the 25 demographic items; and Tables S1.3 and S1.4 present the complete list of 66 scored items with verbatim English wording. Supporting File S2: Psychometric properties, CFA, measurement invariance, and SEM. This file contains the detailed validation of the measurement instruments and the SEM results, including the following: Table S2.1 (psychometric properties: Cronbach’s α, CR, and AVE); Table S2.2 (CFA model fit indices and factor loadings for the JCI patient‐centered standards [six‐factor model, 42 items]); Table S2.3 (CFA model fit indices and factor loadings for the nursing performance model [three‐factor model, 24 items]); Table S2.4 (correlation matrix among JCI patient‐centered standards and nursing performance); Table S2.5 (standardized direct, indirect, and total effects from the SEM); Figure S2.1 (CFA path diagram for the six‐factor JCI patient‐centered standards model); Figure S2.2 (multigroup CFA measurement invariance across public and private hospitals); Figure S2.3 (CFA path diagram for the three‐factor self‐reported nursing performance model); and Figure S2.4 (SEM path diagram showing the second‐order structural model). Supporting File S3: Regression diagnostics, complete regression results, common‐method variance diagnostics, relative weights analysis, and sensitivity analyses. This file contains the following sections: Section A, complete multiple regression results wit [file JONM-2026-8353270-s001.zip › Supplementary_File_S4_STROB_R3_2.docx]

# Supplementary File S4: STROBE Checklist for Cross-Sectional Studies

This supplementary file presents the completed Strengthening the Reporting of Observational Studies in Epidemiology (STROBE) checklist for cross-sectional studies, applied to the manuscript "Association between Joint Commission International Patient-Centered Standards and Self-Reported Nursing Performance in Sana'a, Yemen Hospitals." Page numbers refer to the final revised manuscript.

*Note. *Give information separately for exposed and unexposed groups, where relevant. STROBE checklist items adapted from von Elm E, Altman DG, Egger M, Pocock SJ, Gøtzsche PC, Vandenbroucke JP; STROBE Initiative. The Strengthening the Reporting of Observational Studies in Epidemiology (STROBE) statement: guidelines for reporting observational studies. Lancet. 2007;370(9596):1453–1457.*

**Table S4.1. STROBE checklist for cross-sectional studies.**

| Section/Topic | Item No. | Recommendation | Reported on page(s) |
| --- | --- | --- | --- |
| Title and abstract | 1(a) | Indicate the study's design with a commonly used term in the title or the abstract. | Title (p. 1); Abstract Methods (p. 1) |
|  | 1(b) | Provide in the abstract an informative and balanced summary of what was done and what was found. | Abstract (pp. 1–2) |
| Introduction |  |  |  |
| Background/rationale | 2 | Explain the scientific background and rationale for the investigation being reported. | Introduction, paragraphs 1–3 (pp. 2–3) |
| Objectives | 3 | State specific objectives, including any prespecified hypotheses. | Introduction, final paragraph (p. 3); Study hypotheses (p. 7) |
| Methods |  |  |  |
| Study design | 4 | Present key elements of study design early in the paper. | Methods, "Study design" (p. 3) |
| Setting | 5 | Describe the setting, locations, and relevant dates, including periods of recruitment, exposure, follow-up, and data collection. | Methods, "Setting" (p. 4) |
| Participants | 6(a) | Give the eligibility criteria, and the sources and methods of selection of participants. | Methods, "Participants and sample size" (p. 4); Figure 1 (p. 5) |
| Variables | 7 | Clearly define all outcomes, exposures, predictors, potential confounders, and effect modifiers. Give diagnostic criteria, if applicable. | Methods, "Variables" (p. 6) |
| Data sources/ measurement | 8* | For each variable of interest, give sources of data and details of methods of assessment (measurement). Describe comparability of assessment methods if there is more than one group. | Methods, "Data sources and measurement" (pp. 6–7); Supplementary File S1 |
| Bias | 9 | Describe any efforts to address potential sources of bias. | Methods, "Bias" (p. 7) |
| Study size | 10 | Explain how the study size was arrived at. | Methods, "Participants and sample size" (p. 4) |
| Quantitative variables | 11 | Explain how quantitative variables were handled in the analyses. If applicable, describe which groupings were chosen and why. | Methods, "Statistical methods" (p. 7) |
| Statistical methods | 12(a) | Describe all statistical methods, including those used to control for confounding. | Methods, "Statistical methods" (p. 7) |
|  | 12(b) | Describe any methods used to examine subgroups and interactions. | Methods, "Statistical methods" (p. 7); Sensitivity analyses (p. 13) |
|  | 12(c) | Explain how missing data were addressed. | Methods, "Data sources and measurement" (p. 6); "Statistical methods" (p. 7) |
|  | 12(d) | If applicable, describe analytical methods taking account of sampling strategy. | Methods, "Participants and sample size" (p. 4) |
|  | 12(e) | Describe any sensitivity analyses. | Methods, "Statistical methods" (pp. 7–8); Results, "Sensitivity analyses" (p. 13); Supplementary File S3 |
| Results |  |  |  |
| Participants | 13(a) | Report the numbers of individuals at each stage of the study (e.g., numbers potentially eligible, examined for eligibility, confirmed eligible, included in the study, completing follow-up, and analysed). | Results, "Participants" (p. 8); Table 1 (p. 8); Figure 1 (p. 5) |
|  | 13(b) | Give reasons for non-participation at each stage. | Methods, "Participants and sample size" (p. 4); Figure 1 (p. 5) |
|  | 13(c) | Consider use of a flow diagram. | Figure 1 (p. 5) |
| Descriptive data | 14(a) | Give characteristics of study participants (e.g., demographic, clinical, social) and information on exposures and potential confounders. | Results, "Characteristics of study participants" (p. 9); Table 2 (p. 9) |
|  | 14(b) | Indicate the number of participants with missing data for each variable of interest. | Methods, "Participants and sample size" (p. 4); "Data sources and measurement" (p. 6) |
| Outcome data | 15* | Report numbers of outcome events or summary measures. | Results, Tables 3–4 (p. 10) |
| Main results | 16(a) | Give unadjusted estimates and, if applicable, confounder-adjusted estimates and their precision (e.g., 95% confidence interval). Make clear which confounders were adjusted for and why they were included. | Results, "Testing the study hypotheses" (p. 10); Table 6 (p. 11); Supplementary File S3, Table S3.1 |
|  | 16(b) | Report category boundaries when continuous variables were categorised. | Methods, "Variables" (p. 6); Table 2 (p. 9) |
|  | 16(c) | If relevant, consider translating estimates of relative risk into absolute risk for a meaningful time period. | Not applicable (cross-sectional design without time-to-event outcomes) |
| Other analyses | 17 | Report other analyses done—e.g., analyses of subgroups and interactions, and sensitivity analyses. | Results: CMB/Relative Weights (p. 11); Measurement Invariance (p. 12); Differences by hospital type (pp. 12-13); Sensitivity analyses (p. 13); SEM results (p. 14); Supp Files S2 and S3 |
| Discussion |  |  |  |
| Key results | 18 | Summarise key results with reference to study objectives. | Discussion, paragraph 1 (p. 14) |
| Limitations | 19 | Discuss limitations of the study, taking into account sources of potential bias or imprecision. Discuss both direction and magnitude of any potential bias. | Discussion, "Limitations" (pp. 17–18) |
| Interpretation | 20 | Give a cautious overall interpretation of results considering objectives, limitations, multiplicity of analyses, results from similar studies, and other relevant evidence. | Discussion (pp. 14–17); Conclusion (p. 18) |
| Generalisability | 21 | Discuss the generalisability (external validity) of the study results. | Discussion, "Limitations" (paragraph 7, p. 18); Conclusion (p. 18) |
| Other information |  |  |  |
| Funding | 22 | Give the source of funding and the role of the funders for the present study and, if applicable, for the original study on which the present article is based. | Declarations, "Funding" (p. 19) |

*Note. STROBE, Strengthening the Reporting of Observational Studies in Epidemiology. Page numbers refer to the final revised manuscript.*
